# Supplementary material for: Therapeutic Cancer Vaccination with Immunopeptidomics-Discovered Antigens Confers Protective Antitumor Efficacy
Source: Cancers (Basel). 2021 Jul 7;13(14):3408. doi: 10.3390/cancers13143408 (PMC8306067; doi:10.3390/cancers13143408)
Supplement: Supplementary file 1 [file cancers-13-03408-s001.zip › cancers-1267045-supplementary.pdf]

Supplementary Materials: Therapeutic Cancer Vaccination with Immunopeptidomics-Discovered Antigens Confers Protective Antitumor Efficacy

Karita Peltonen, Sara Feola, Husen M. Umer , Jacopo Chiaro, Georgios Mermelekas, Erkko Ylösmäki, Sari Pesonen, Rui M. M. Branca, Janne Lehtiö and Vincenzo Cerullo

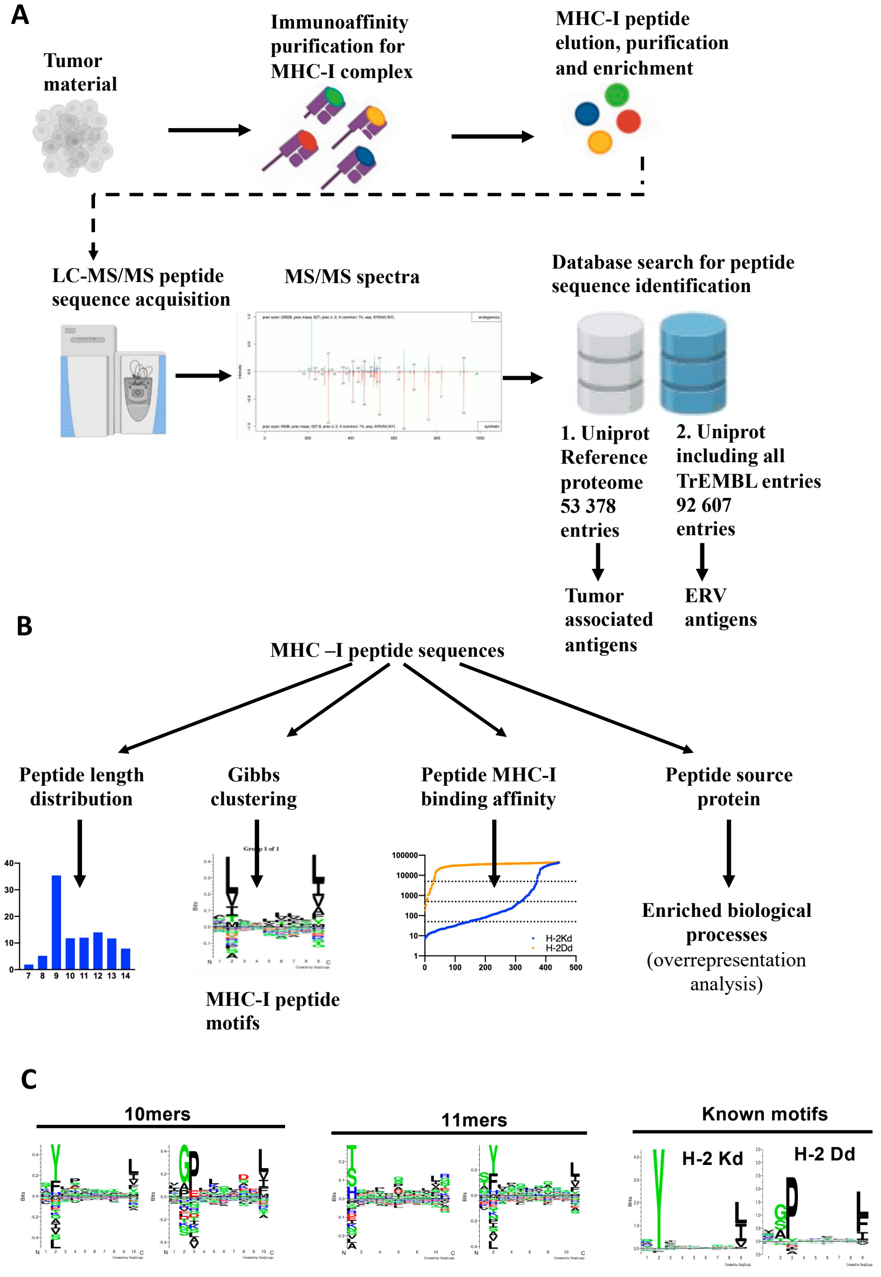

Figure S1. (**A**) Overal workflow for the discovery of MHC-I presented antigens. Tumor associated antigens were identified by searching the mouse reference proteome and ERV antigen by extending the search to include all TrEMBL entries. (**B**) Bioinformatic analysis workflow. (**C**) Gibbs clustering for the identified 10mers and 11mers and known H-2K^{d} and H-2D^{d} ligand binding motifs based on eluted, naturally presented ligand data.

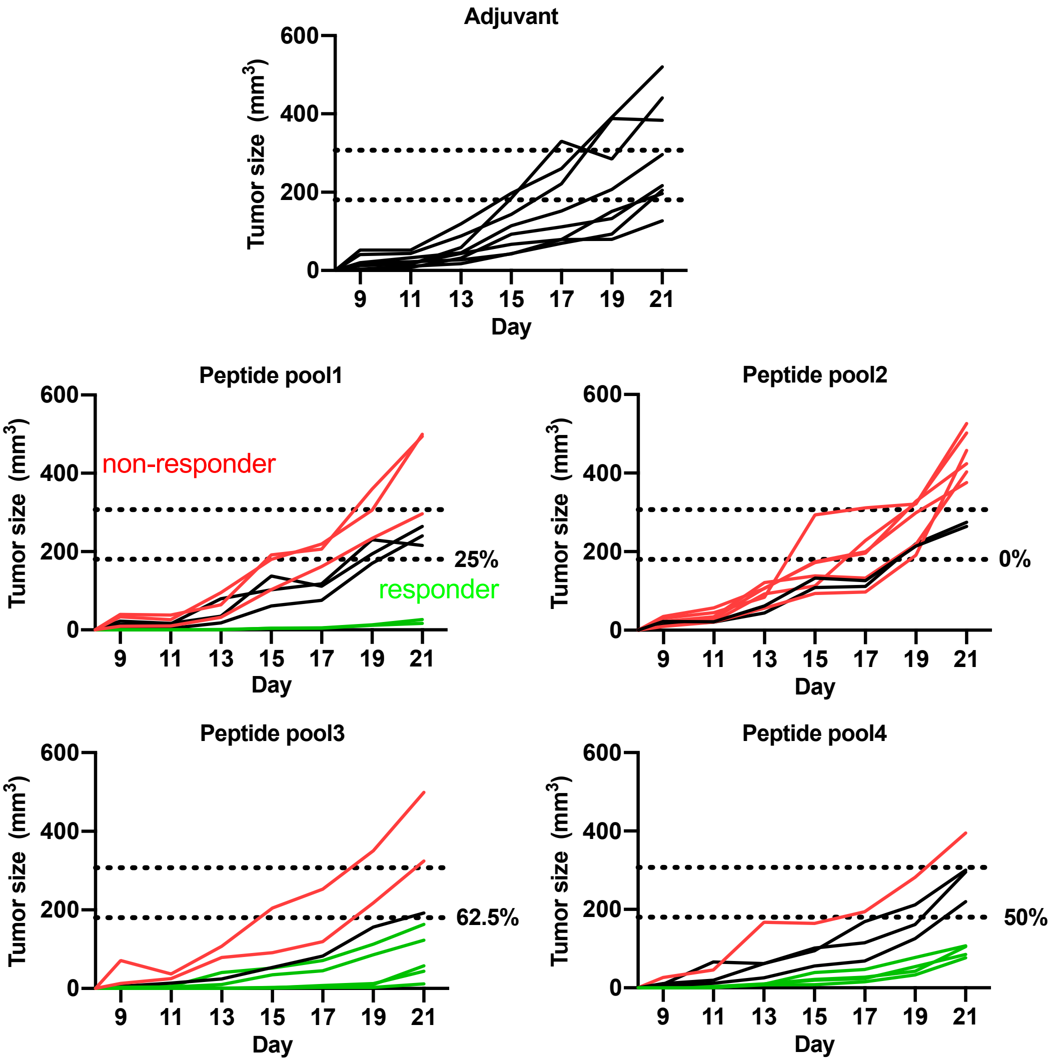

Figure S2. Individual tumor growth curves. Dotted lines represent threshold for responders and non-responders. The responder and non-responder mice are highlighted in green and red, respectively, and the percentage of responding mice within the group is given. $N = 8$ for each group.

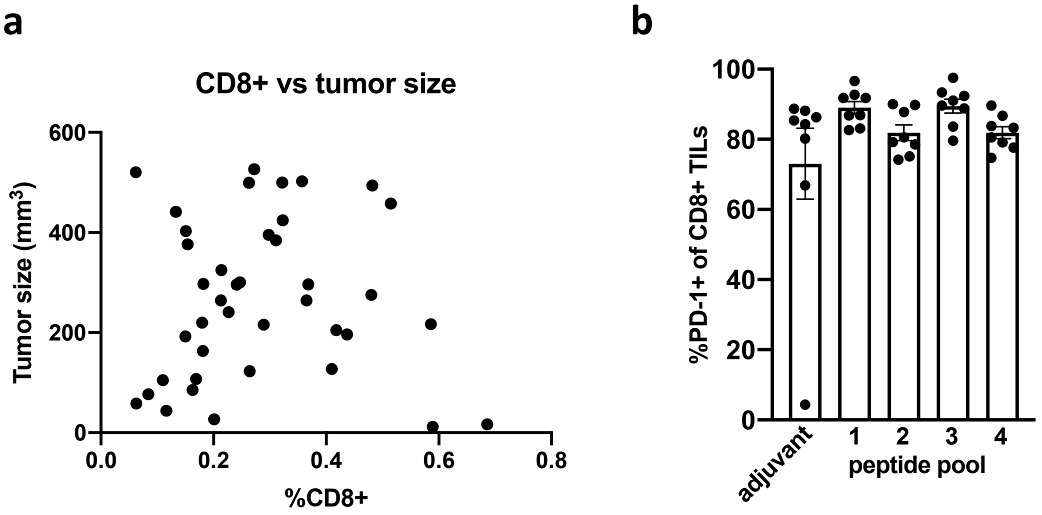

Figure S3. Analysis of tumor infiltrating CD8+ T-cells. (**A**) Correlation analysis of tumor size at endpoint (day 21) vs. percentage of tumor infiltrating CD8+ cells. (**B**) Percentage of PD-1 positive CD8+ cells in tumors.

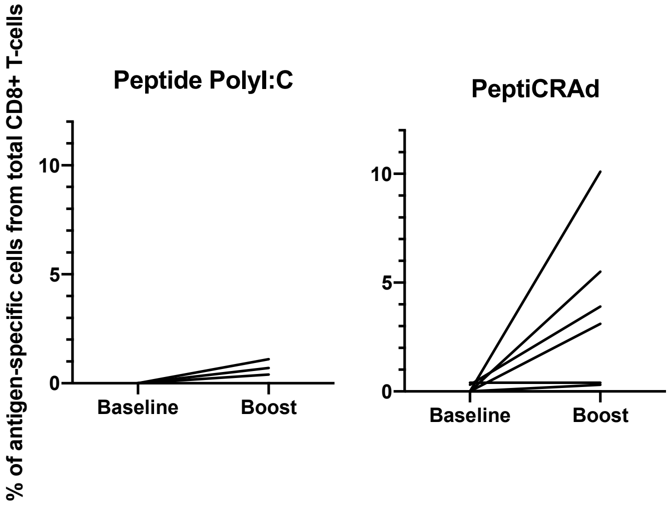

Figure S4. Comparison of the vaccination platforms for stimulation of antigen-specific T-cells. Tumor bearing mice were vaccinated with NY-ESO-1 peptide (91-110aa) either intradermally together with Poly I:C as an adjuvant (Peptide PolyI:C) or intratumorally as PeptiCRAd complexation (PeptiCRAd). Three priming injections were given on consecutive days followed by one booster injection after 7 days. Blood samples were drawn for the flow cytometric assessment of antigen-specific CD8+ T-cells using tetramer labeling against NY-ESO-1 at baseline (4 days prior first priming) and two weeks after the booster (boost). As animal model humanized mice (NOD/Shi-scid/IL-2Rγnull (NOG) mice (Taconic) engrafted with cord blood-derived CD34+ hematopoietic stem cells) were used. Engraftment was allowed to establish for 15 weeks prior to monitoring of engraftment level, and animals with a humanization rate equal or above 30% were included in the study.

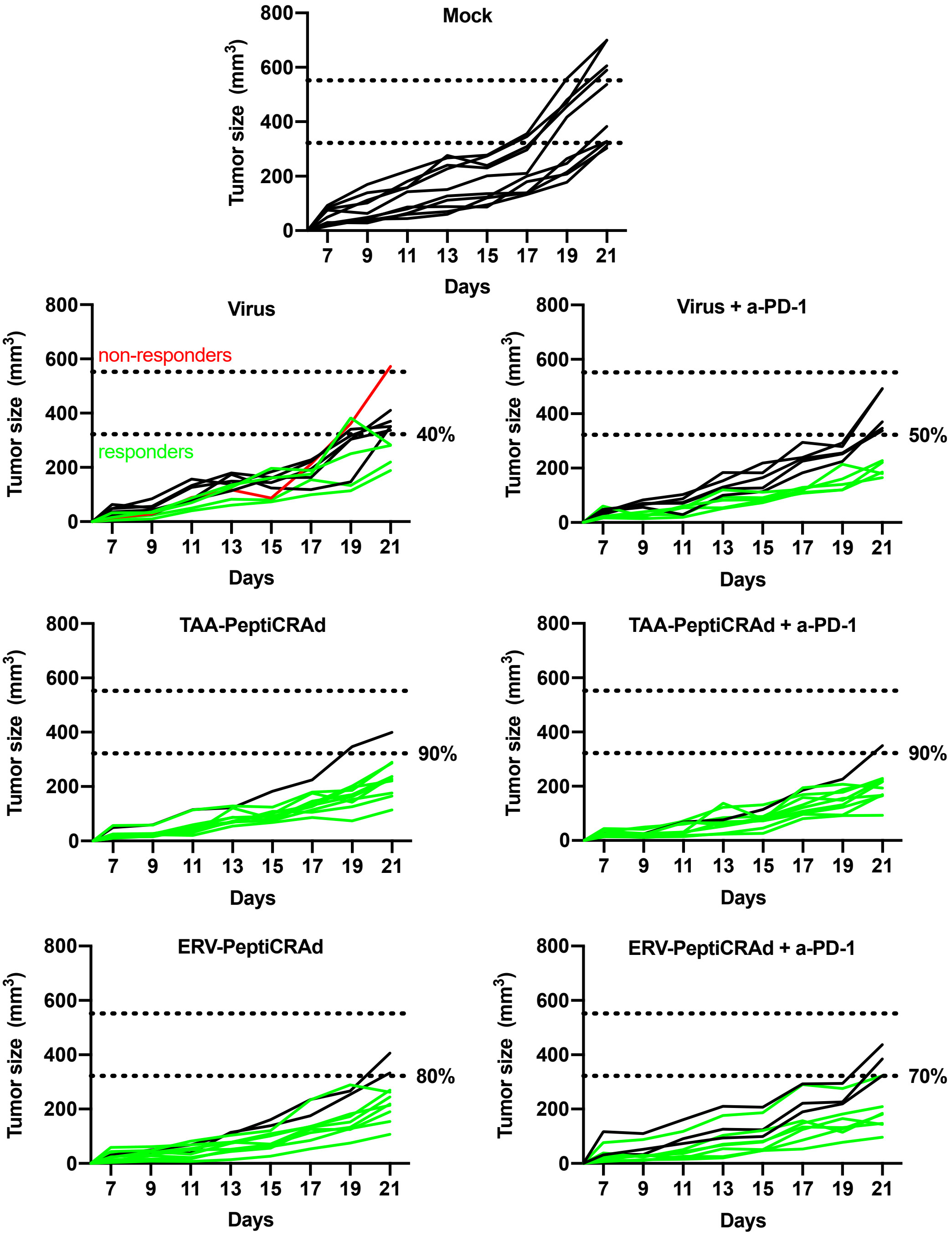

Figure S5. Individual tumor growth curves for the therapeutic vaccination experiments using PeptiCRAd platform. Dotted lines represent threshold for responders and non-responders. The responder and non-responder mice are highlighted in green and red, respectively, and the percentage of responding mice within the group is given. Statistical analysis of tumor growth curves was performed using two-way Anova with Tukey's multiple comparison. There was statistical difference between mock and each other treatment group (adjusted P-value < 0.0001); Virus group and each other treatment group except Virus + anti-PD1 (adjusted P-value 0.013 or lower); and between Virus + anti-PD-1 and TAA-PeptiCRAd + anti-PD1 (adjusted P-value 0.0012). N = 10 for each group.
