# Supplementary material for: Therapeutic Cancer Vaccination with Immunopeptidomics-Discovered Antigens Confers Protective Antitumor Efficacy
Source: Cancers (Basel). 2021 Jul 7;13(14):3408. doi: 10.3390/cancers13143408 (PMC8306067; doi:10.3390/cancers13143408)
Supplement: Supplementary file 1 [file cancers-13-03408-s001.zip › Supplementary_data file_2.pdf]

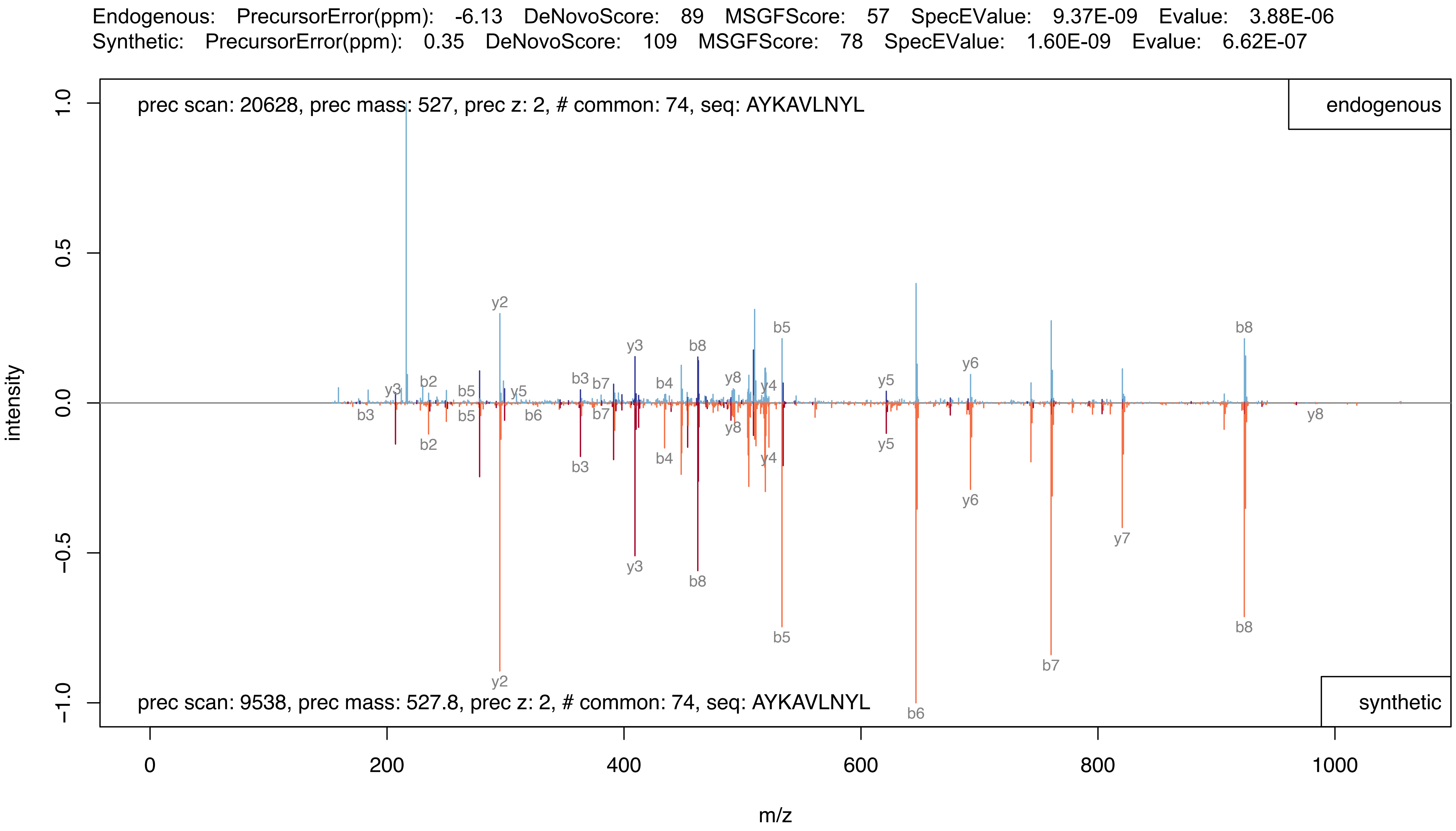
Endogenous: PrecursorError(ppm): -6.13 DeNovoScore: 89 MSGFScore: 57 SpecEValue: 9.37E-09 Evalue: 3.88E-06
Synthetic: PrecursorError(ppm): 0.35 DeNovoScore: 109 MSGFScore: 78 SpecEValue: 1.60E-09 Evalue: 6.62E-07
prec scan: 20628, prec mass: 527, prec z: 2, # common: 74, seq: AYKAVLNYL
endogenous
prec scan: 9538, prec mass: 527.8, prec z: 2, # common: 74, seq: AYKAVLNYL
synthetic
intensity
m/z
1.0
0.5
0.0
−0.5
−1.0
0
200
400
600
800
1000

Endogenous: PrecursorError(ppm): -6.15 DeNovoScore: 50 MSGFScore: 43 SpecEValue: 1.60E-11 Evalue: 6.64E-09
Synthetic: PrecursorError(ppm): 1.27 DeNovoScore: 99 MSGFScore: 99 SpecEValue: 3.13E-12 Evalue: 1.29E-09

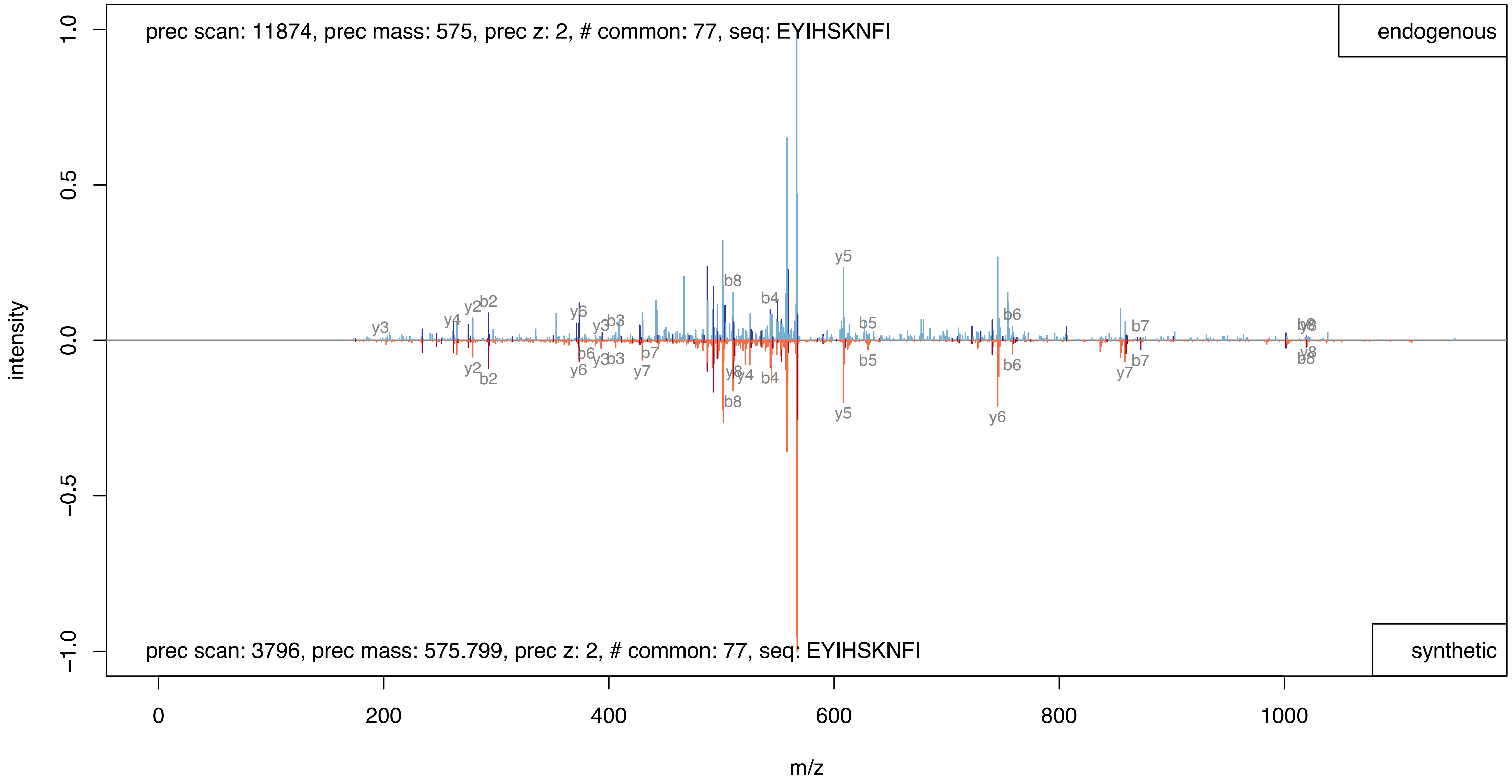

Endogenous: PrecursorError(ppm): -3.13 DeNovoScore: 144 MSGFScore: 90 SpecEValue: 2.10E-08 Evalue: 8.70E-06
Synthetic: PrecursorError(ppm): 0.35 DeNovoScore: 144 MSGFScore: 100 SpecEValue: 8.07E-09 Evalue: 3.34E-06

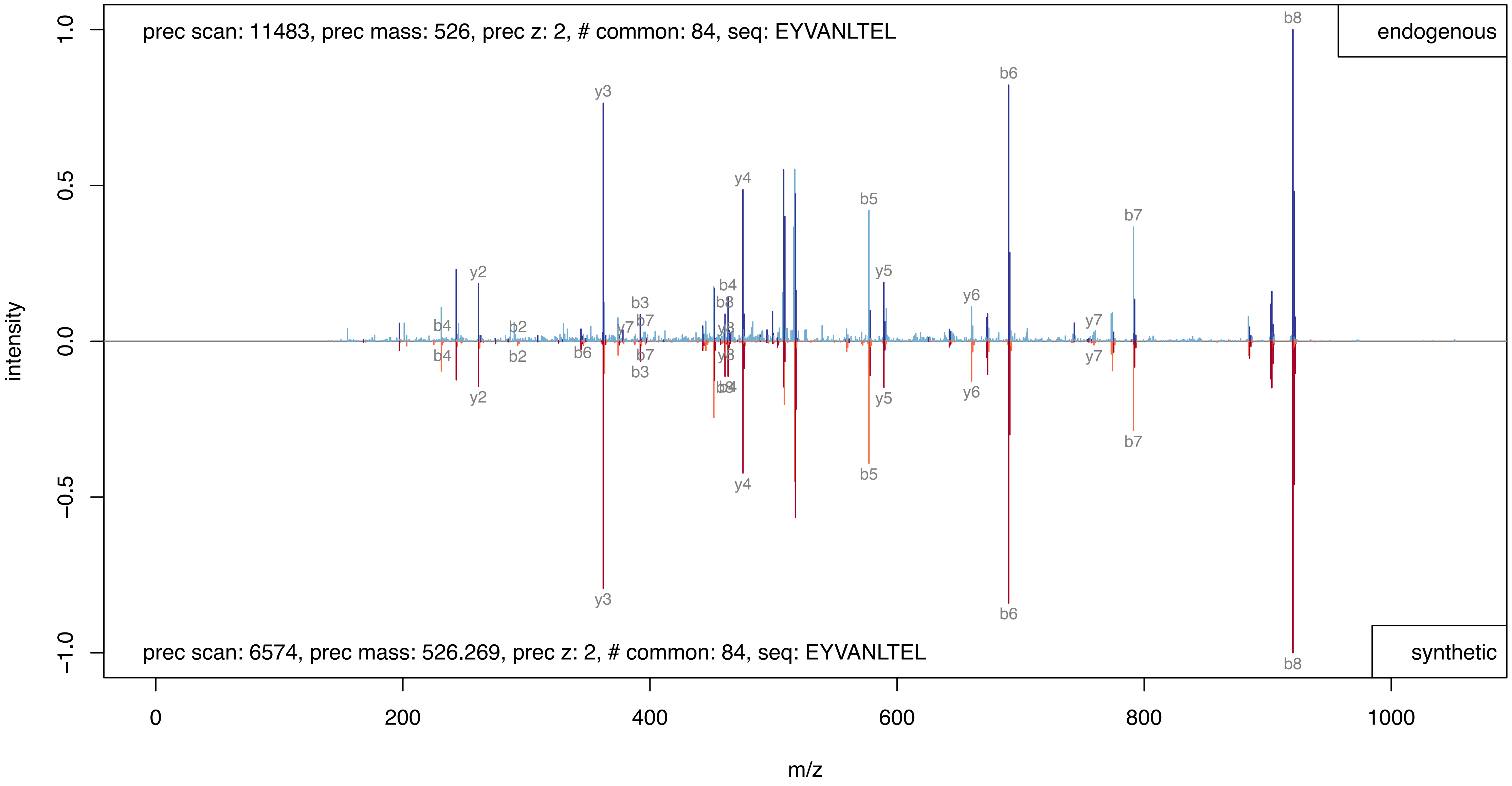

Endogenous: PrecursorError(ppm): -6.28 DeNovoScore: 72 MSGFScore: 24 SpecEValue: 2.11E-05 Evalue: 8.72E-03
Synthetic: PrecursorError(ppm): 0.64 DeNovoScore: 52 MSGFScore: 15 SpecEValue: 4.43E-06 Evalue: 1.83E-03

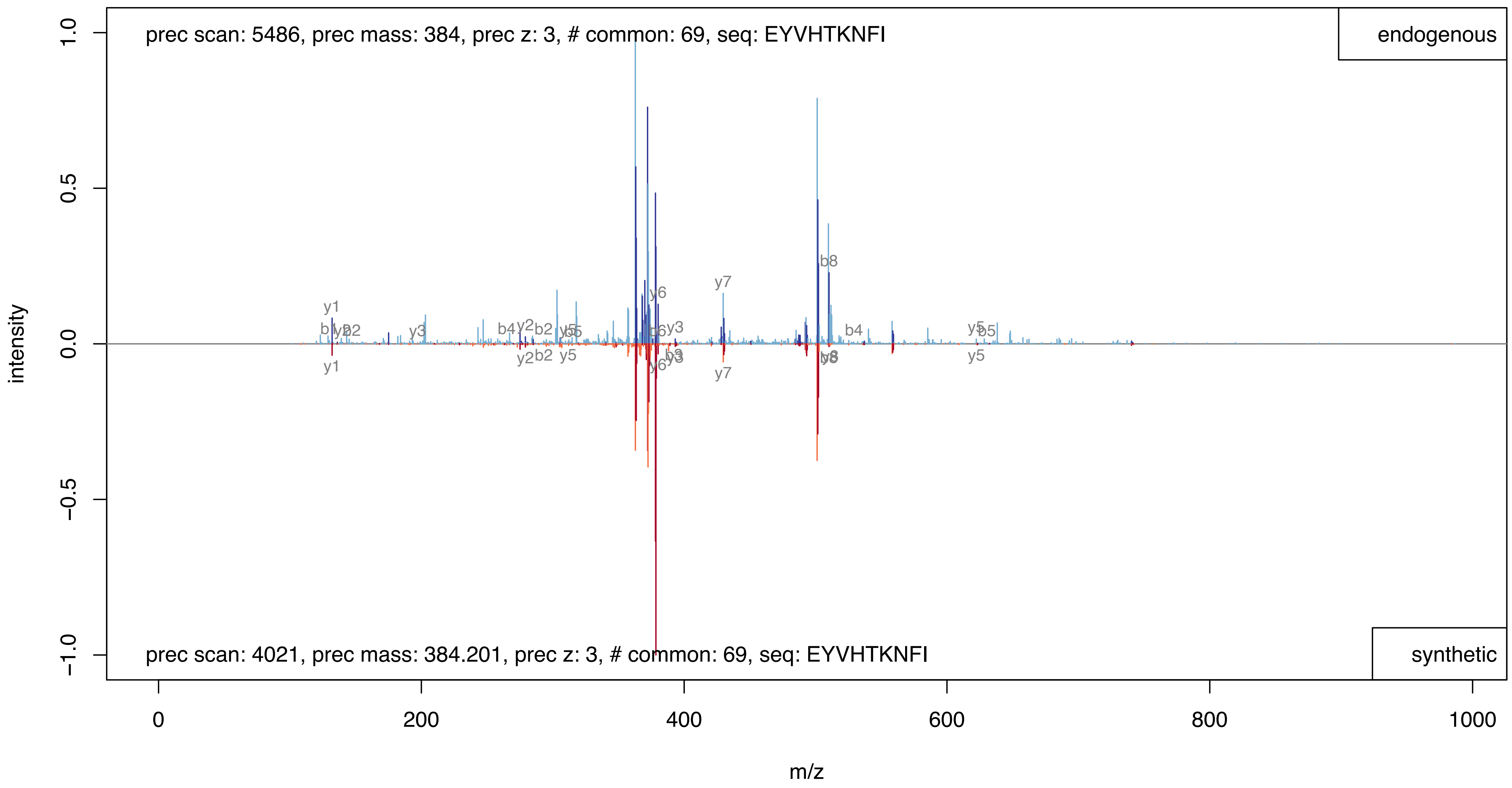

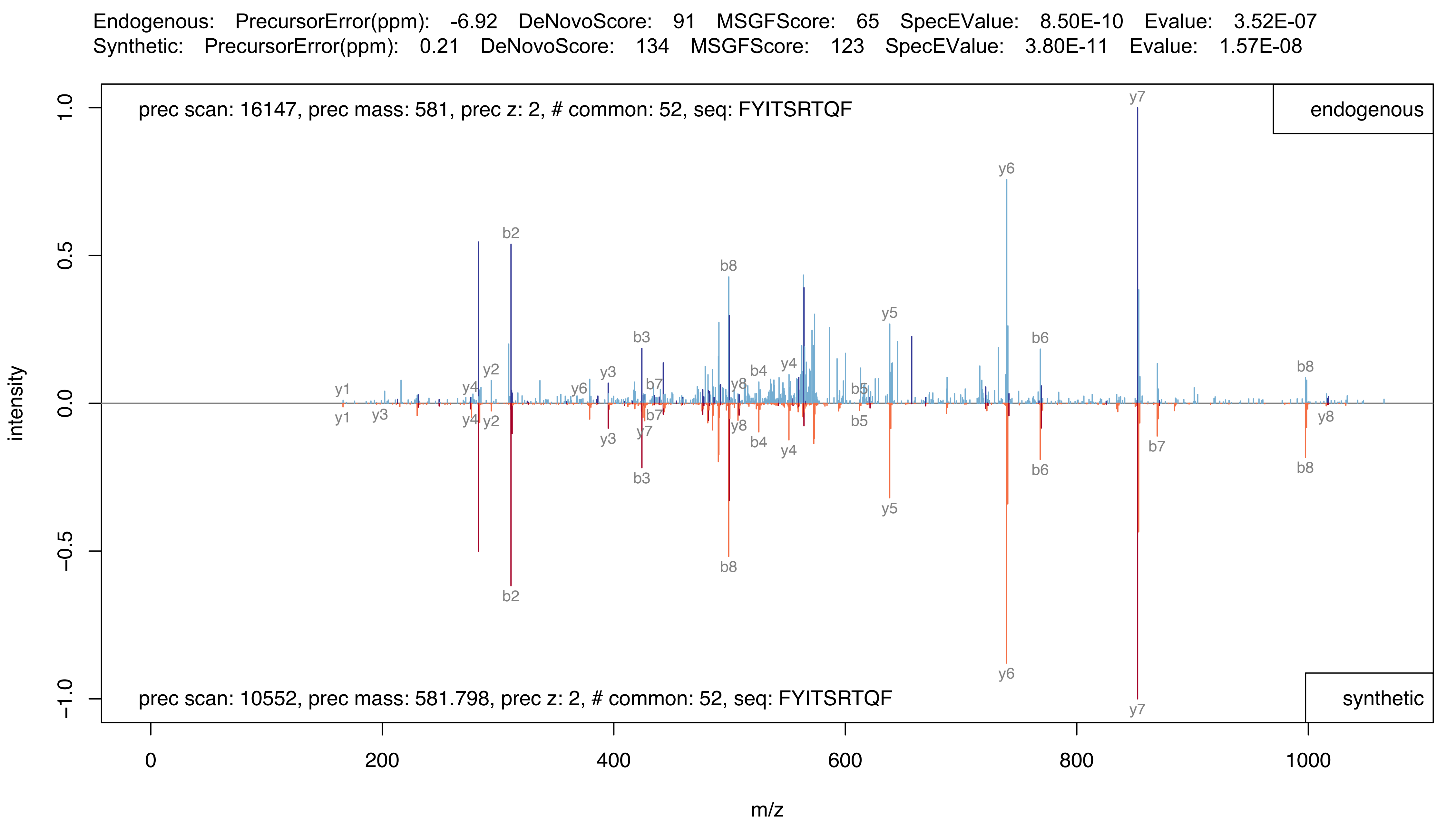
Endogenous: PrecursorError(ppm): -6.92 DeNovoScore: 91 MSGFScore: 65 SpecEValue: 8.50E-10 Evalue: 3.52E-07
Synthetic: PrecursorError(ppm): 0.21 DeNovoScore: 134 MSGFScore: 123 SpecEValue: 3.80E-11 Evalue: 1.57E-08
prec scan: 16147, prec mass: 581, prec z: 2, # common: 52, seq: FYITSRTQF
endogenous
prec scan: 10552, prec mass: 581.798, prec z: 2, # common: 52, seq: FYITSRTQF
synthetic
intensity
m/z
1.0
0.5
0.0
−0.5
−1.0
0
200
400
600
800
1000
y1
y2
y3
y4
y5
y6
y7
y8
b2
b3
b4
b5
b6
b7
b8

Endogenous: PrecursorError(ppm): -8.76 DeNovoScore: 28 MSGFScore: 11 SpecEValue: 1.75E-09 Evalue: 7.23E-07
Synthetic: PrecursorError(ppm): -1.56 DeNovoScore: 58 MSGFScore: 46 SpecEValue: 6.81E-11 Evalue: 2.82E-08

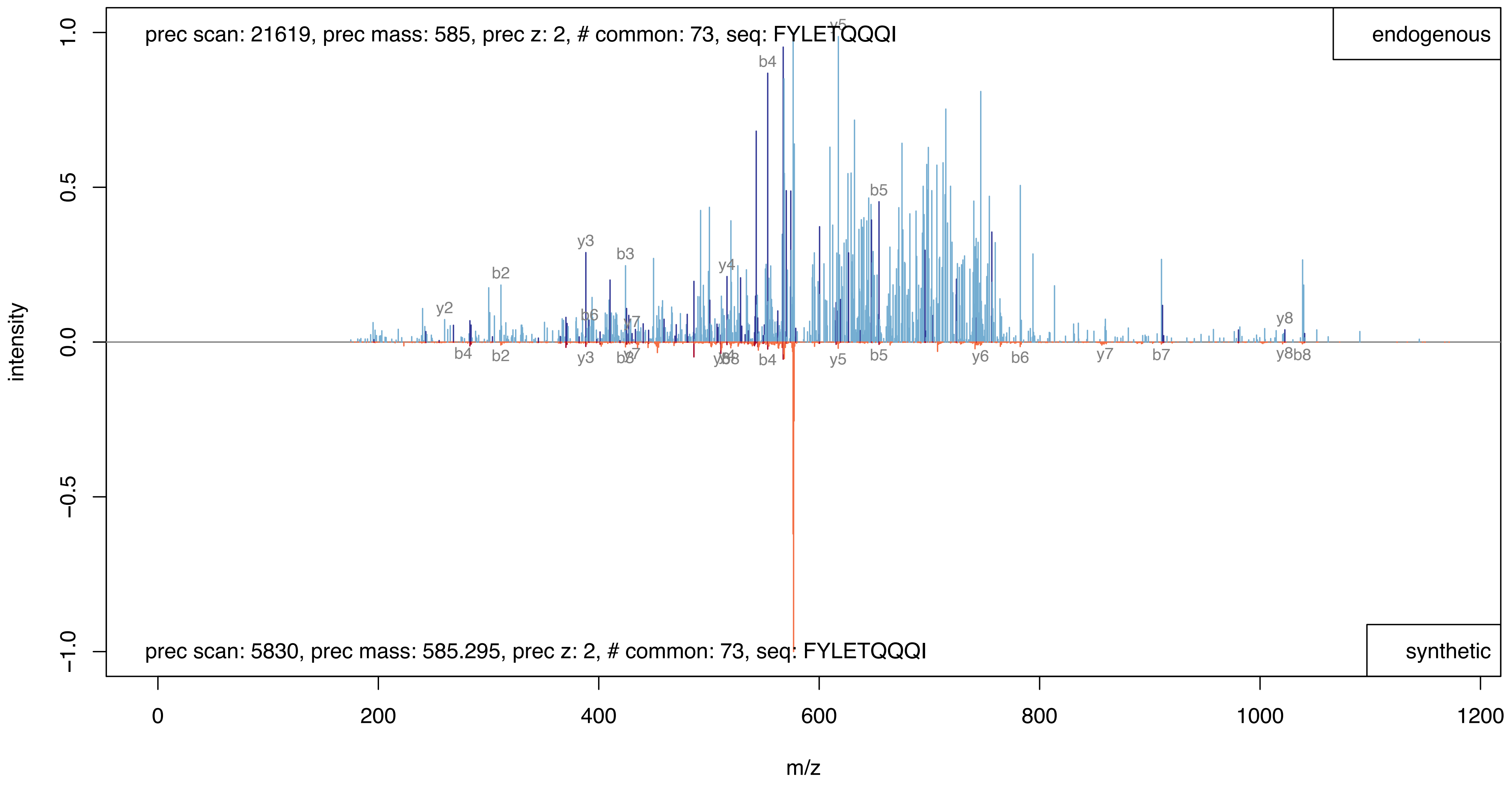

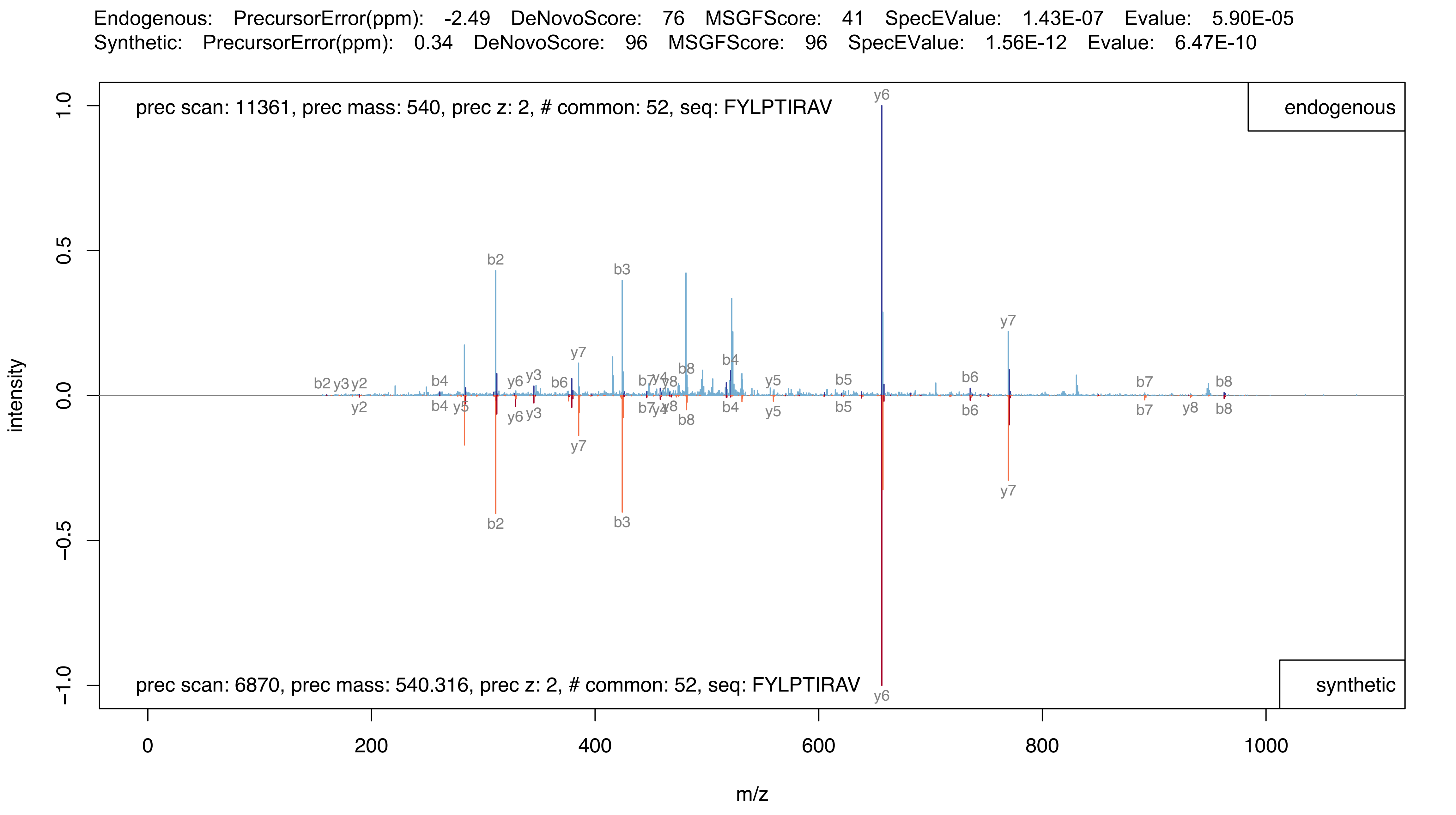
Endogenous: PrecursorError(ppm): -2.49 DeNovoScore: 76 MSGFScore: 41 SpecEValue: 1.43E-07 Evalue: 5.90E-05
Synthetic: PrecursorError(ppm): 0.34 DeNovoScore: 96 MSGFScore: 96 SpecEValue: 1.56E-12 Evalue: 6.47E-10
prec scan: 11361, prec mass: 540, prec z: 2, # common: 52, seq: FYLPTIRAV
endogenous
prec scan: 6870, prec mass: 540.316, prec z: 2, # common: 52, seq: FYLPTIRAV
synthetic
intensity
m/z
1.0
0.5
0.0
−0.5
−1.0
0
200
400
600
800
1000
y6
b2
b3
y7
b8
b4
b5
b6
b7
y5
y8

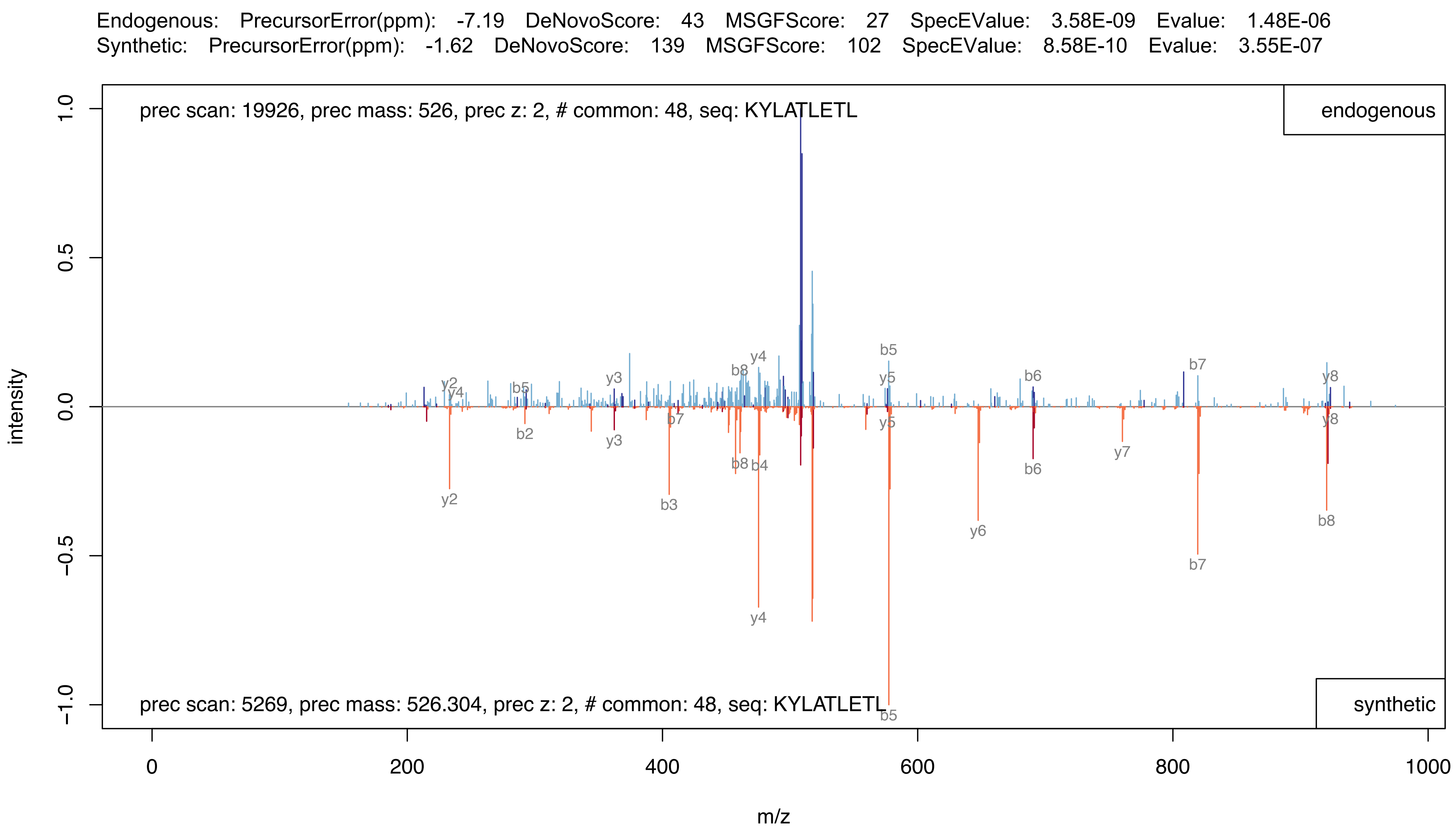
Endogenous: PrecursorError(ppm): -7.19 DeNovoScore: 43 MSGFScore: 27 SpecEValue: 3.58E-09 Evalue: 1.48E-06
Synthetic: PrecursorError(ppm): -1.62 DeNovoScore: 139 MSGFScore: 102 SpecEValue: 8.58E-10 Evalue: 3.55E-07
prec scan: 19926, prec mass: 526, prec z: 2, # common: 48, seq: KYLATLETL
endogenous
prec scan: 5269, prec mass: 526.304, prec z: 2, # common: 48, seq: KYLATLETL
synthetic
intensity
m/z
1.0
0.5
0.0
−0.5
−1.0
0
200
400
600
800
1000
y2
y4
b5
y3
b8
y4
b5
y5
b6
b7
y8
b2
b7
y3
b8
b4
y5
b6
y7
y8
y2
b3
y6
b7
b8
y4
b5

Endogenous: PrecursorError(ppm): -4.41 DeNovoScore: 54 MSGFScore: 7 SpecEValue: 6.68E-07 Evalue: 2.76E-04
Synthetic: PrecursorError(ppm): 0.18 DeNovoScore: 114 MSGFScore: 71 SpecEValue: 6.77E-10 Evalue: 2.80E-07

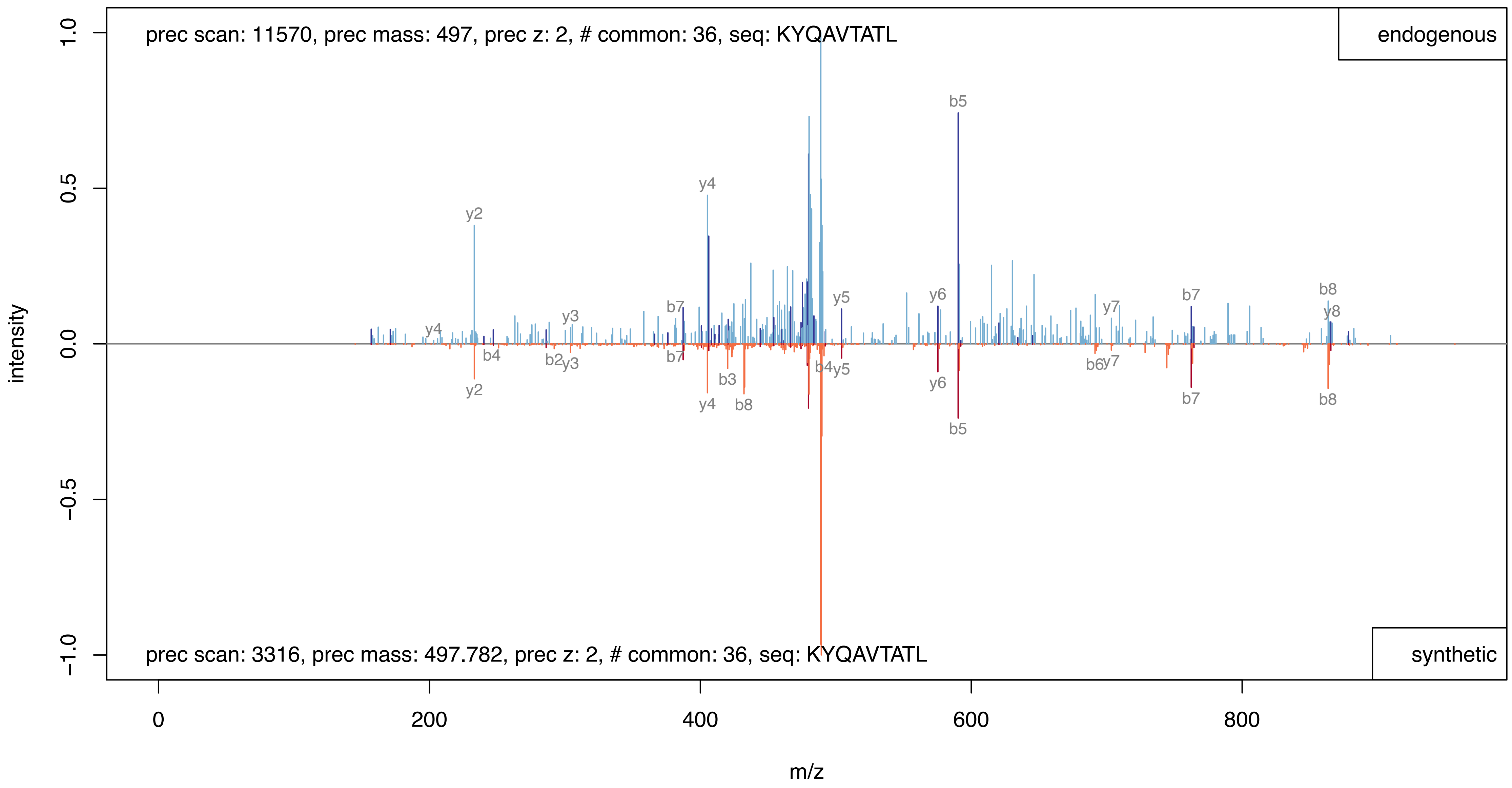

Endogenous: PrecursorError(ppm): -5.54 DeNovoScore: 95 MSGFScore: 19 SpecEValue: 1.55E-05 Evalue: 6.40E-03
Synthetic: PrecursorError(ppm): -0.57 DeNovoScore: 119 MSGFScore: 61 SpecEValue: 9.68E-08 Evalue: 4.01E-05

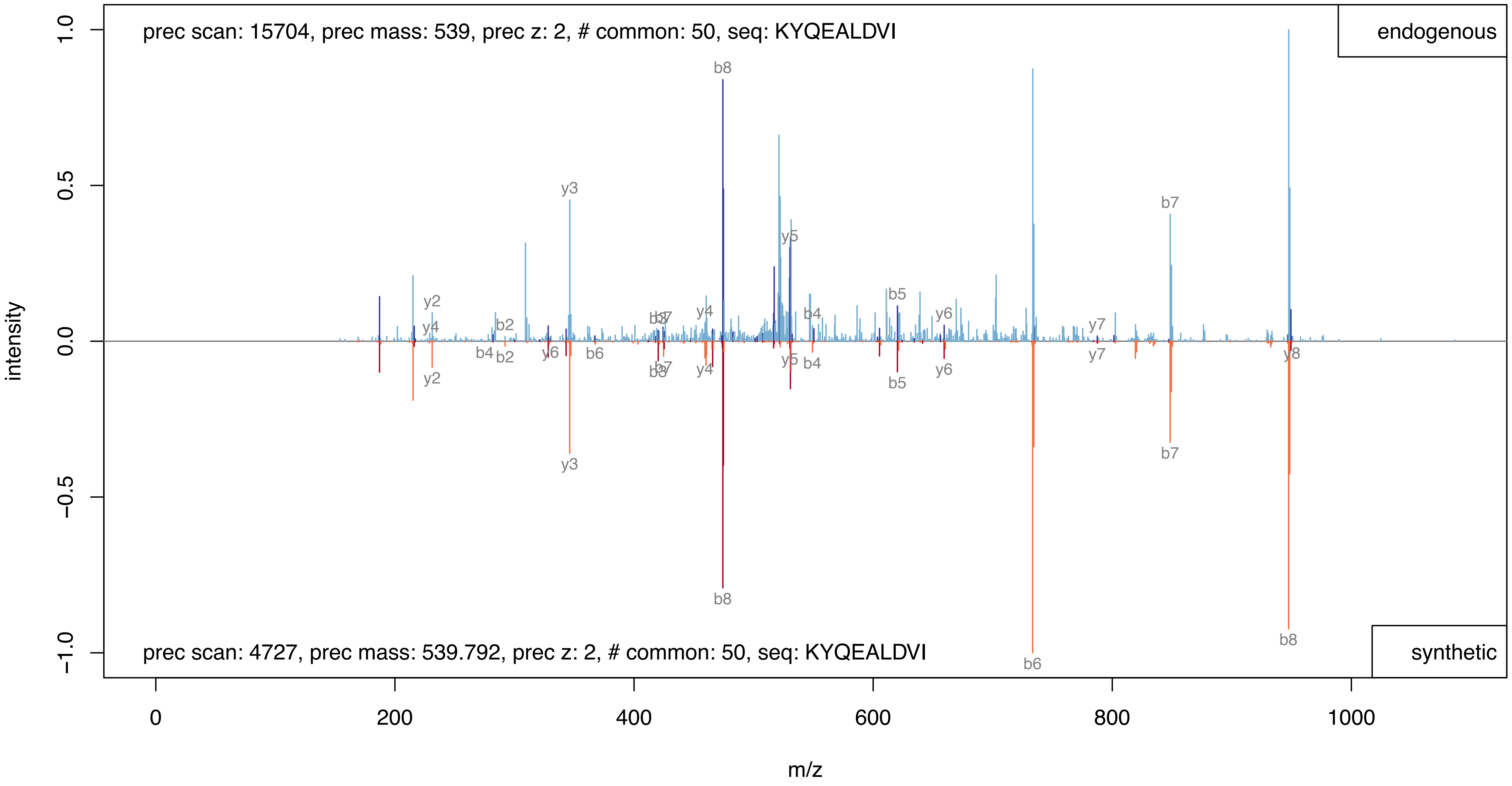

Endogenous: PrecursorError(ppm): -6.06 DeNovoScore: 108 MSGFScore: 57 SpecEValue: 1.29E-07 Evalue: 5.35E-05
Synthetic: PrecursorError(ppm): 0.69 DeNovoScore: 154 MSGFScore: 97 SpecEValue: 2.30E-08 Evalue: 9.54E-06

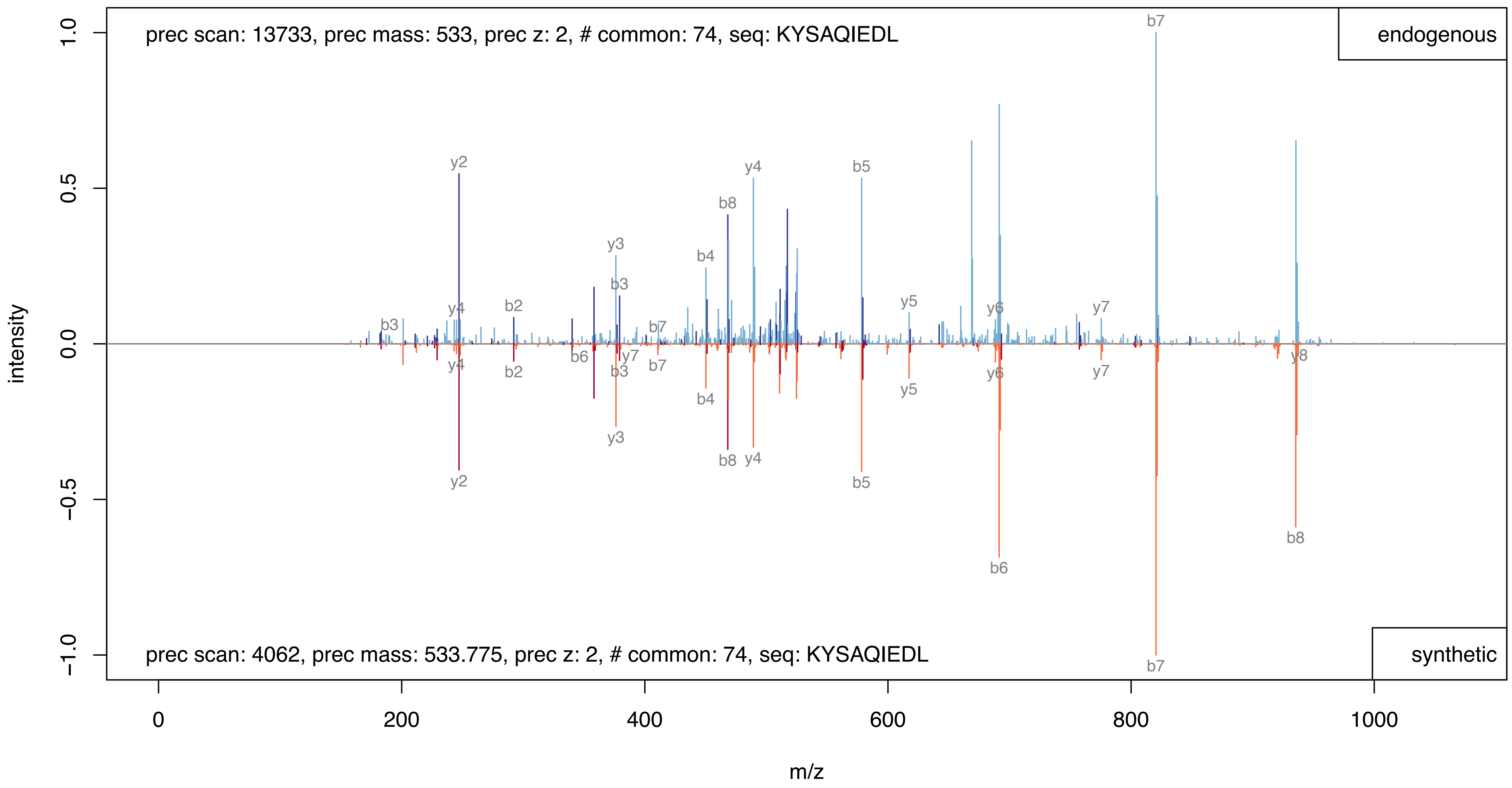

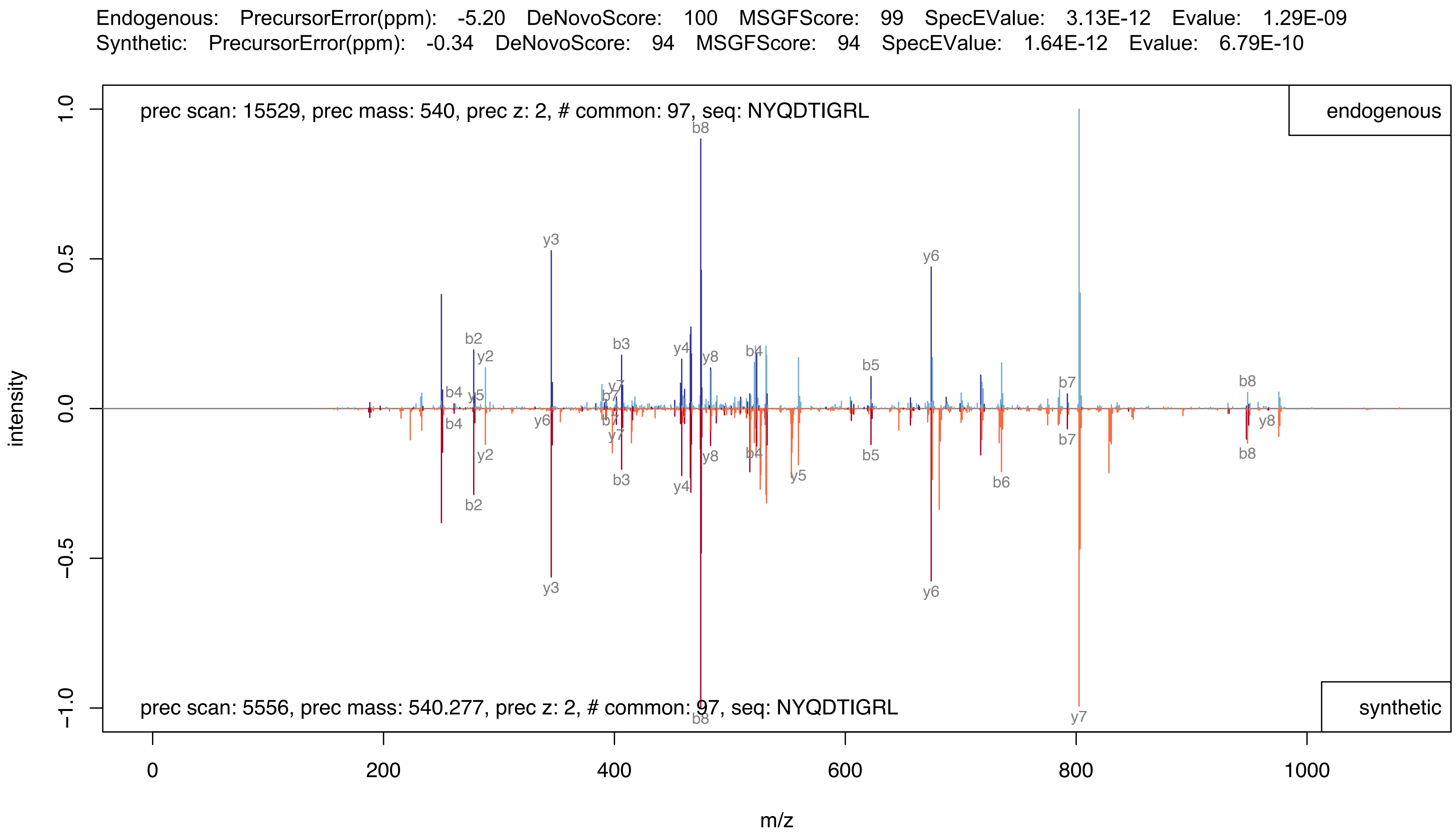

Endogenous: PrecursorError(ppm): -5.20 DeNovoScore: 100 MSGFScore: 99 SpecEValue: 3.13E-12 Evalue: 1.29E-09
Synthetic: PrecursorError(ppm): -0.34 DeNovoScore: 94 MSGFScore: 94 SpecEValue: 1.64E-12 Evalue: 6.79E-10
prec scan: 15529, prec mass: 540, prec z: 2, # common: 97, seq: NYQDTIGRL
endogenous
prec scan: 5556, prec mass: 540.277, prec z: 2, # common: 97, seq: NYQDTIGRL
synthetic
intensity
m/z

Endogenous: PrecursorError(ppm): -5.00 DeNovoScore: 98 MSGFScore: 67 SpecEValue: 1.93E-09 Evalue: 7.99E-07
Synthetic: PrecursorError(ppm): -0.83 DeNovoScore: 99 MSGFScore: 80 SpecEValue: 1.72E-10 Evalue: 7.10E-08

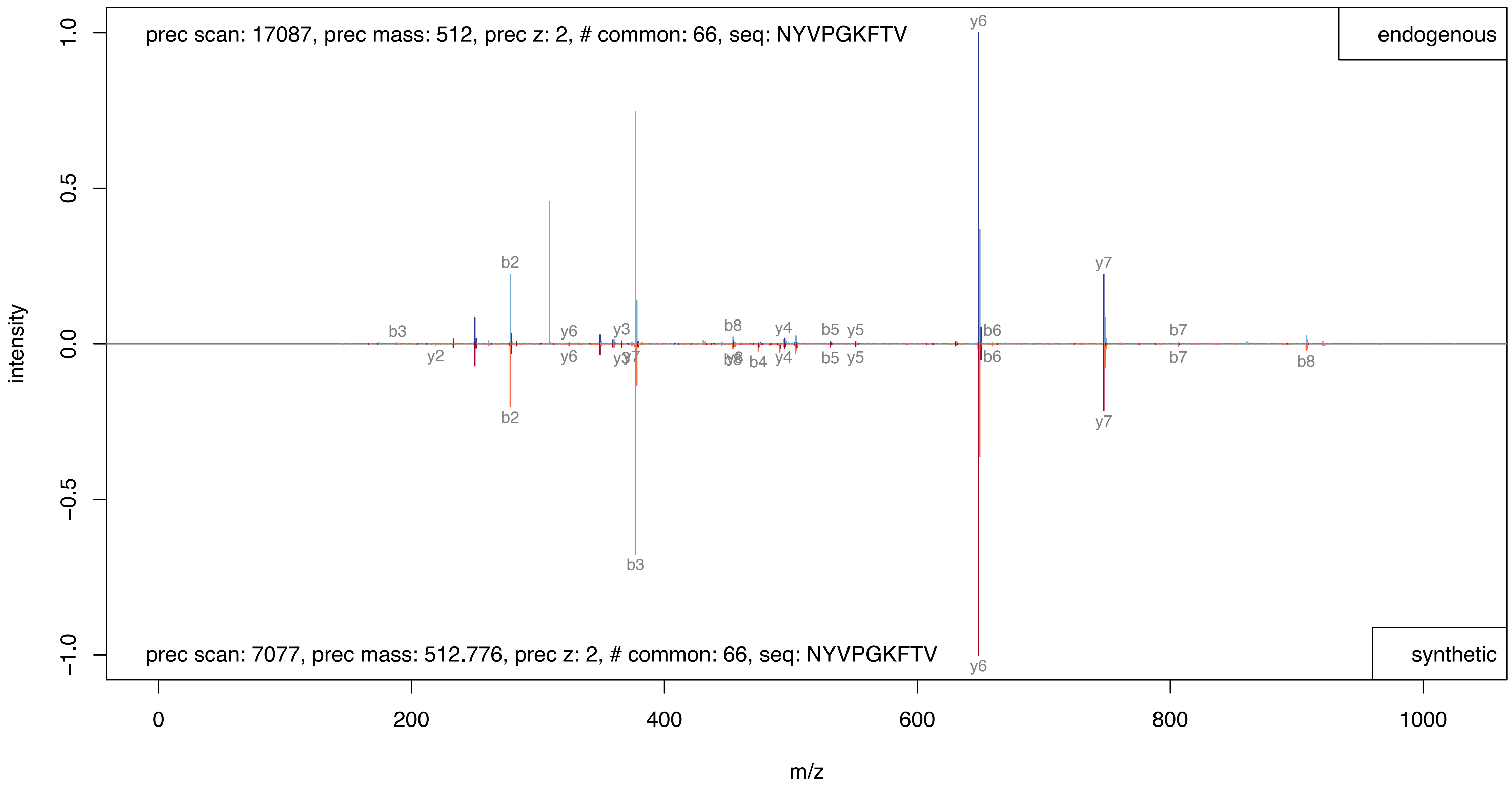

Endogenous: PrecursorError(ppm): -5.60 DeNovoScore: 87 MSGFScore: 43 SpecEValue: 8.31E-09 Evalue: 3.44E-06
Synthetic: PrecursorError(ppm): -0.18 DeNovoScore: 92 MSGFScore: 51 SpecEValue: 5.49E-10 Evalue: 2.27E-07

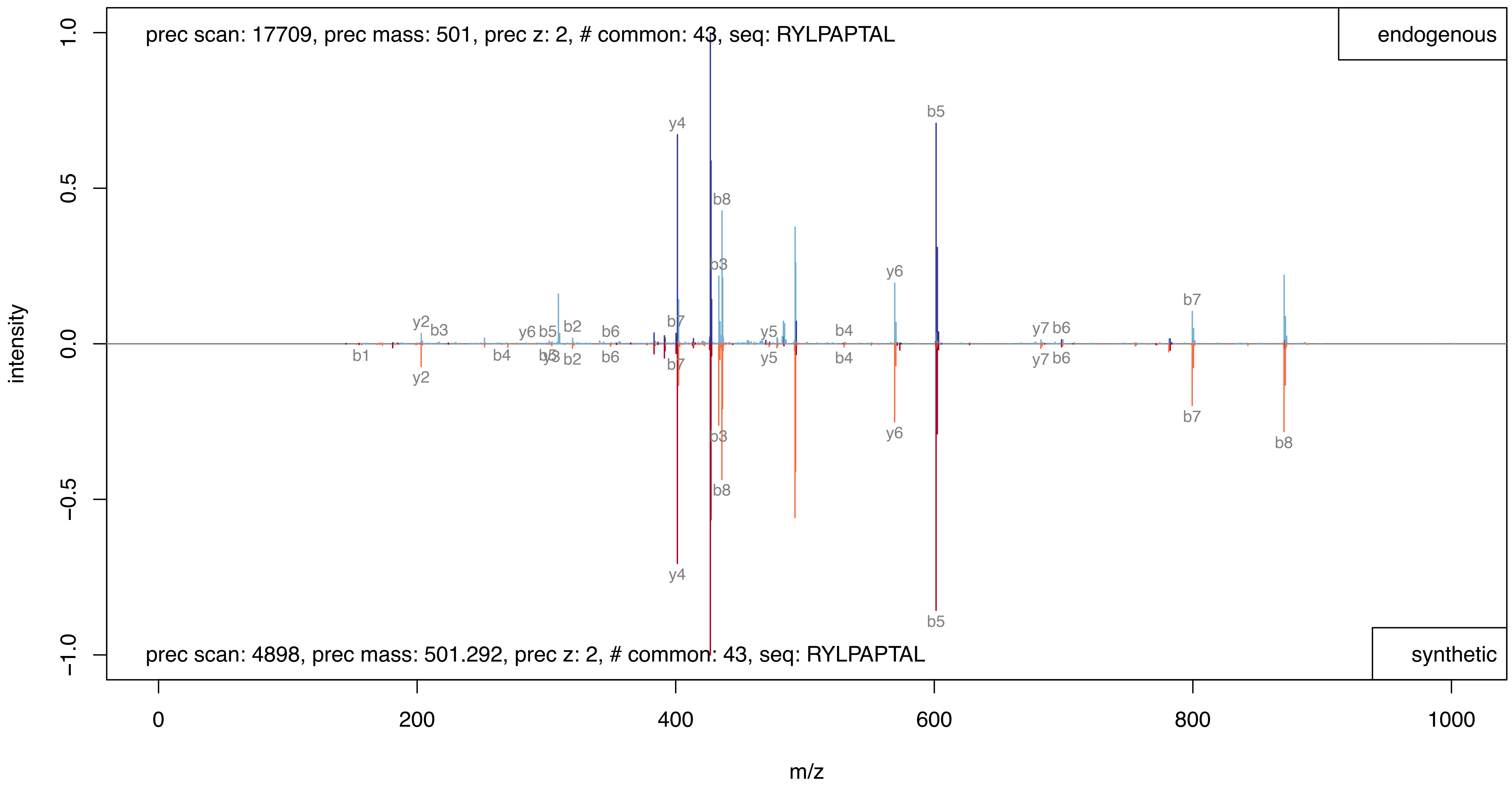

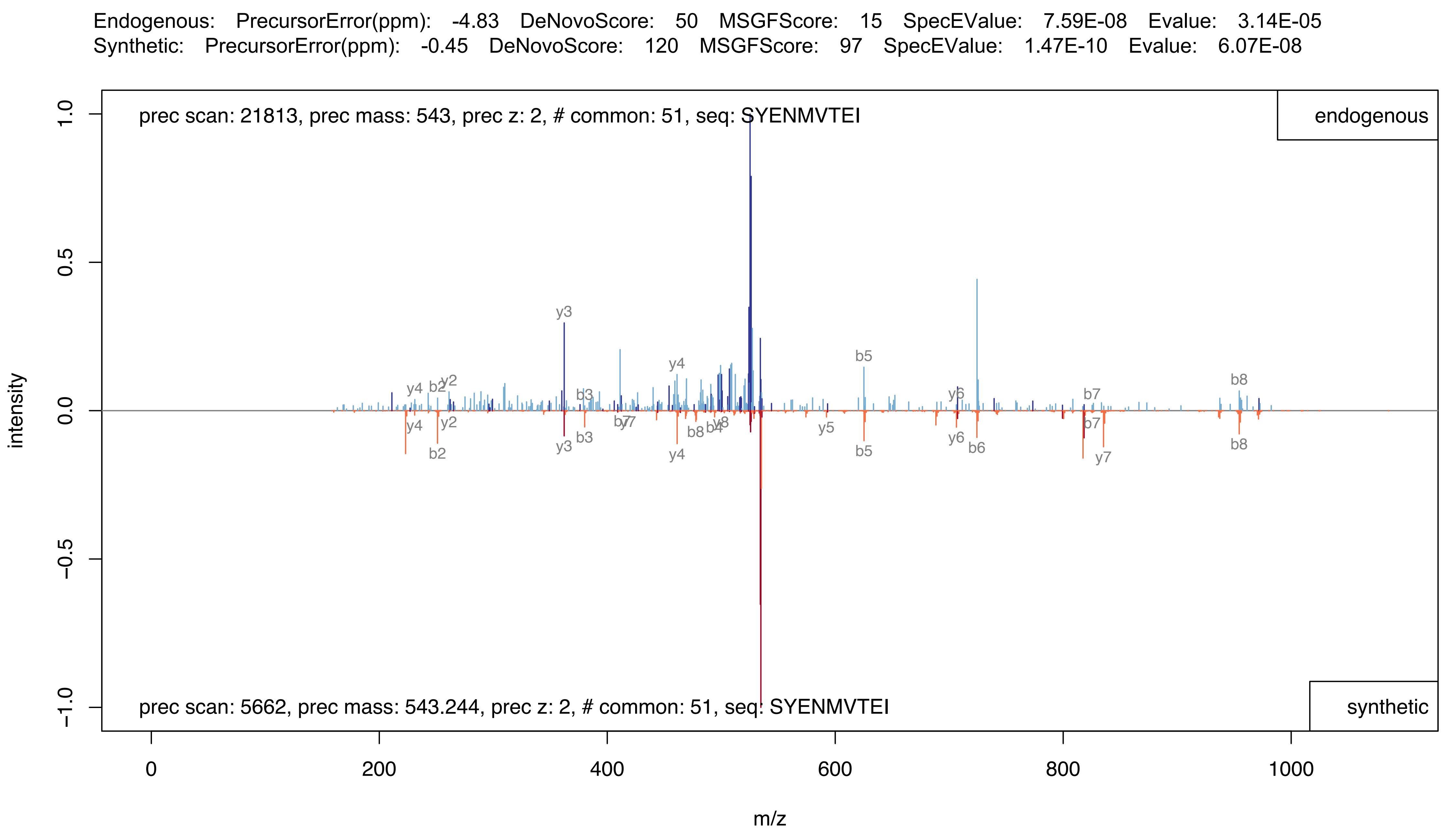
Endogenous: PrecursorError(ppm): -4.83 DeNovoScore: 50 MSGFScore: 15 SpecEValue: 7.59E-08 Evalue: 3.14E-05
Synthetic: PrecursorError(ppm): -0.45 DeNovoScore: 120 MSGFScore: 97 SpecEValue: 1.47E-10 Evalue: 6.07E-08
prec scan: 21813, prec mass: 543, prec z: 2, # common: 51, seq: SYENMVTEI
endogenous
prec scan: 5662, prec mass: 543.244, prec z: 2, # common: 51, seq: SYENMVTEI
synthetic
intensity
m/z
1.0
0.5
0.0
−0.5
−1.0
0
200
400
600
800
1000
y3
y4
b2
y2
b3
b5
b6
b7
b8
y5
y6
y7
y8
b4

Endogenous: PrecursorError(ppm): -2.09 DeNovoScore: 92 MSGFScore: 75 SpecEValue: 8.87E-10 Evalue: 3.67E-07
Synthetic: PrecursorError(ppm): -1.54 DeNovoScore: 112 MSGFScore: 107 SpecEValue: 4.69E-12 Evalue: 1.94E-09

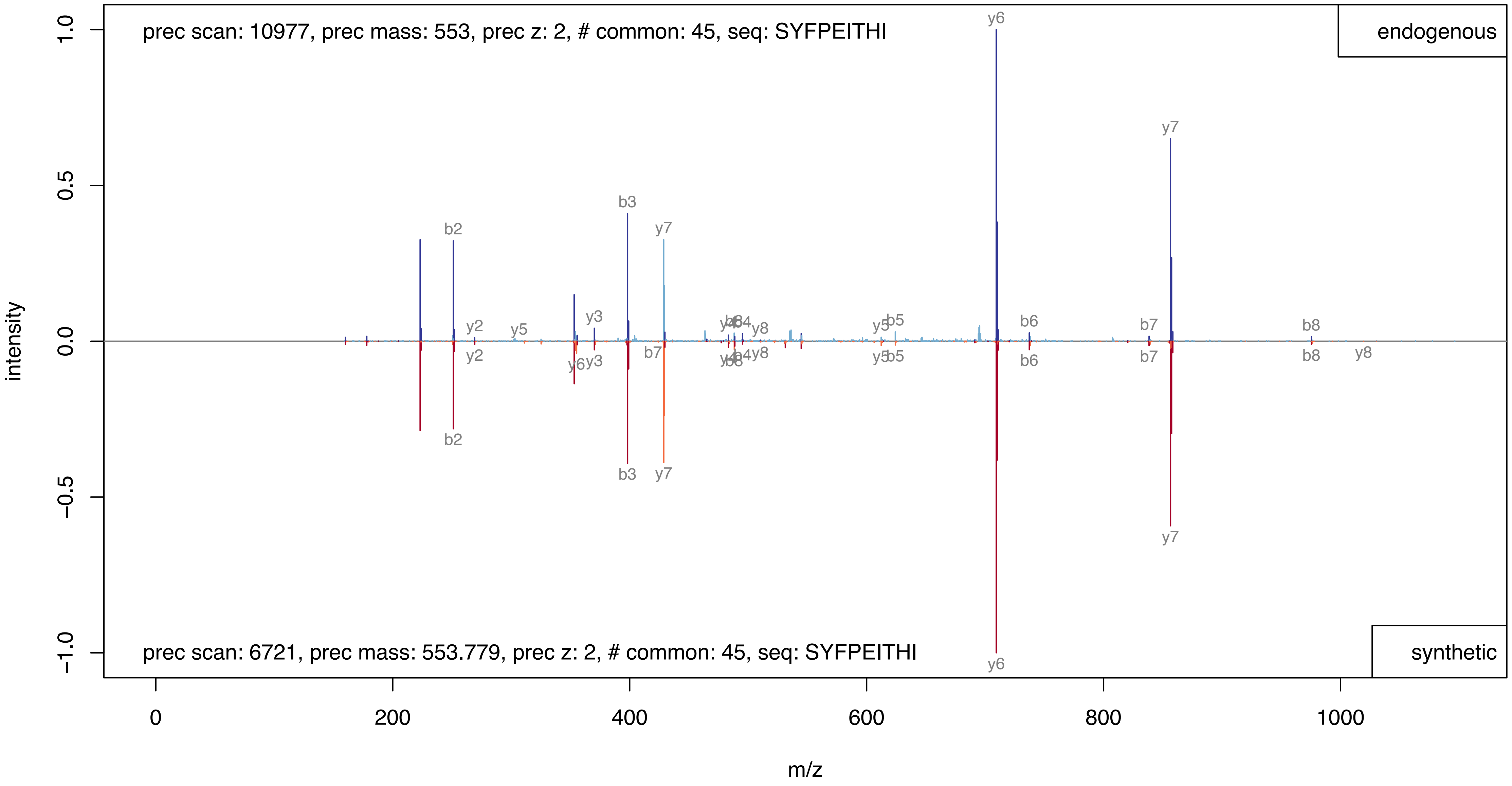

Endogenous: PrecursorError(ppm): -6.31 DeNovoScore: 100 MSGFScore: 34 SpecEValue: 5.20E-07 Evalue: 2.15E-04
Synthetic: PrecursorError(ppm): -0.99 DeNovoScore: 79 MSGFScore: 36 SpecEValue: 9.99E-09 Evalue: 4.13E-06

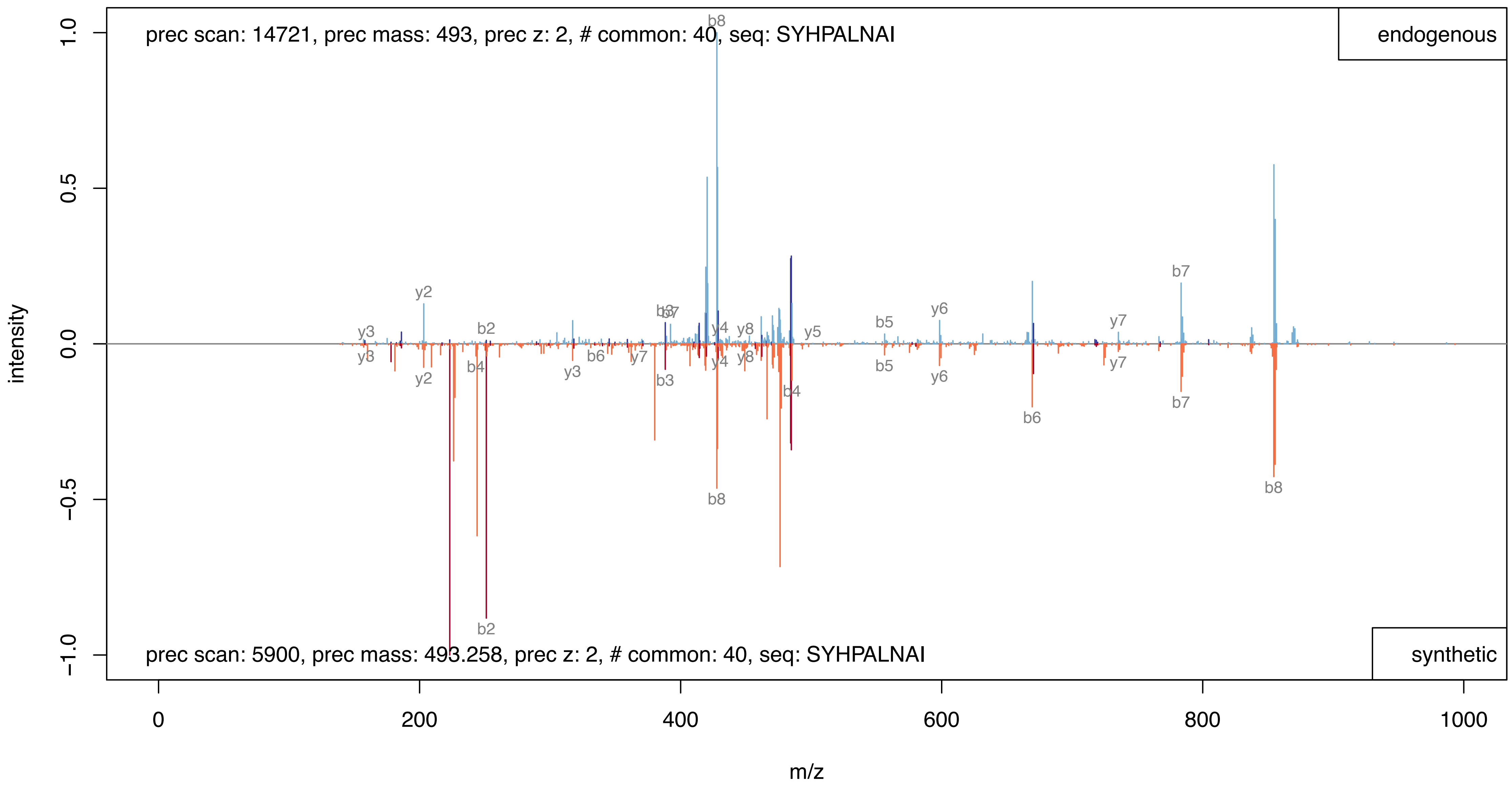

Endogenous: PrecursorError(ppm): -8.01 DeNovoScore: 74 MSGFScore: 28 SpecEValue: 3.56E-07 Evalue: 1.47E-04
Synthetic: PrecursorError(ppm): -1.21 DeNovoScore: 114 MSGFScore: 73 SpecEValue: 2.84E-09 Evalue: 1.18E-06

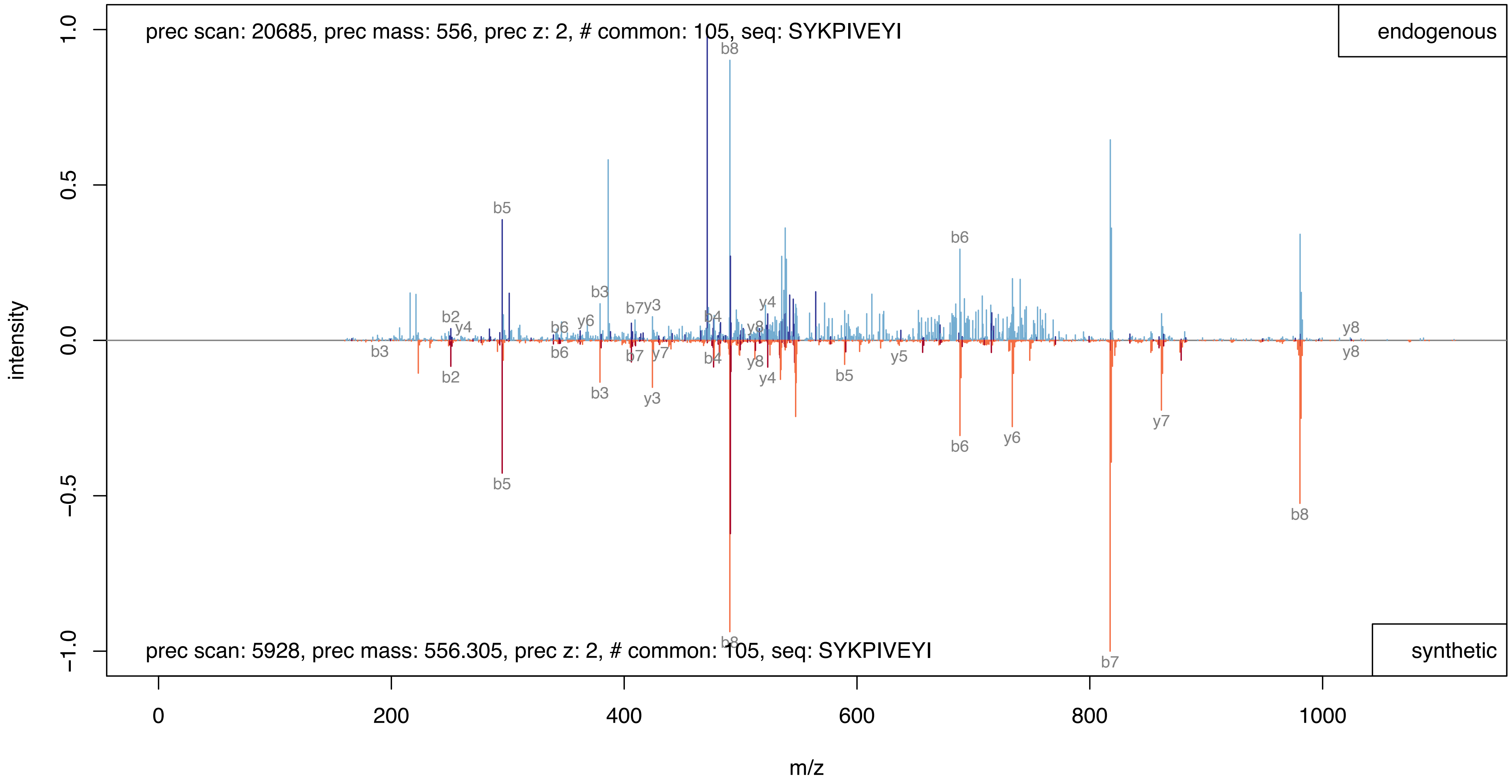

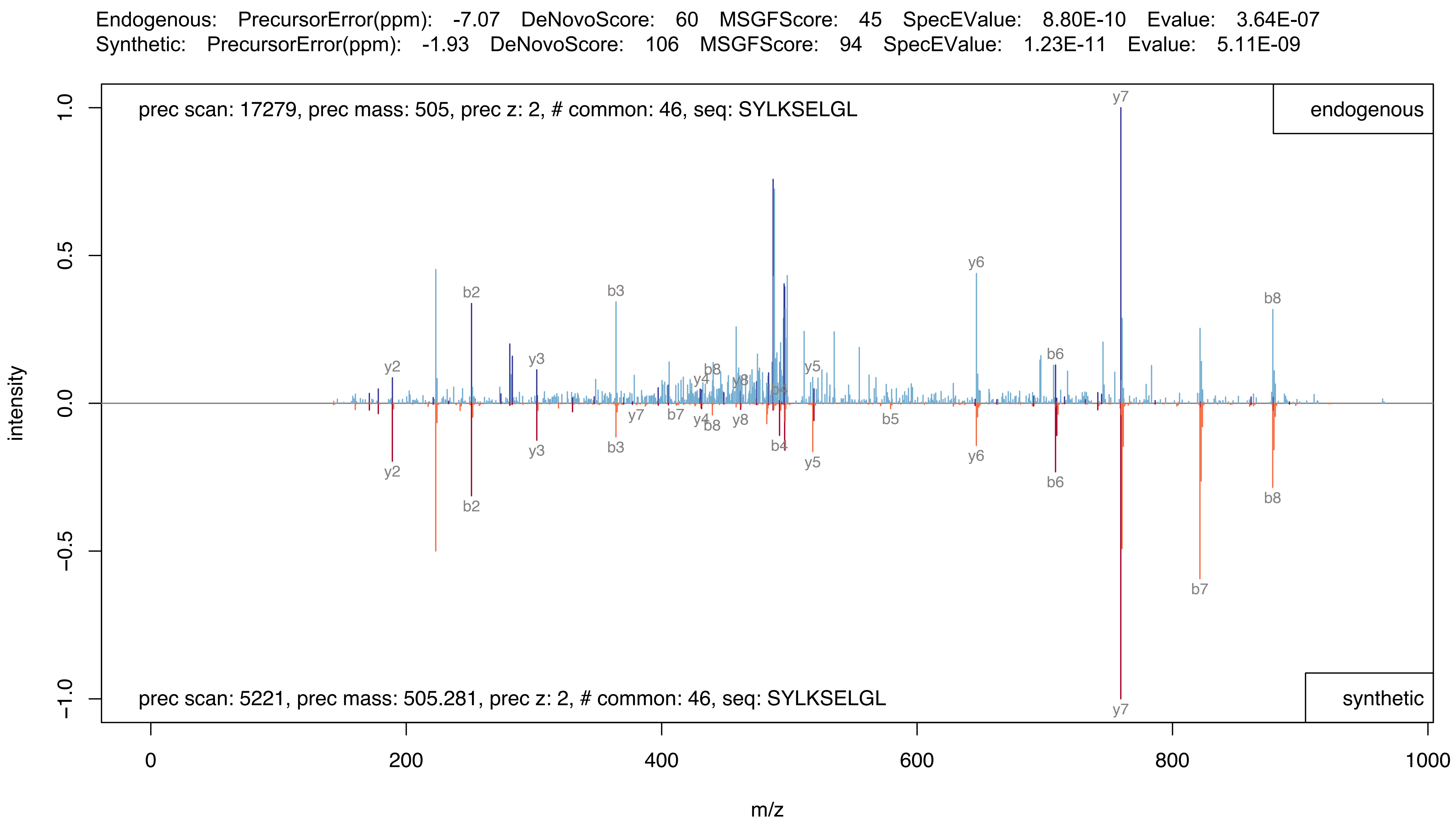
Endogenous: PrecursorError(ppm): -7.07 DeNovoScore: 60 MSGFScore: 45 SpecEValue: 8.80E-10 Evalue: 3.64E-07
Synthetic: PrecursorError(ppm): -1.93 DeNovoScore: 106 MSGFScore: 94 SpecEValue: 1.23E-11 Evalue: 5.11E-09
prec scan: 17279, prec mass: 505, prec z: 2, # common: 46, seq: SYLKSELGL
endogenous
prec scan: 5221, prec mass: 505.281, prec z: 2, # common: 46, seq: SYLKSELGL
synthetic
intensity
m/z
1.0
0.5
0.0
−0.5
−1.0
0
200
400
600
800
1000
y2
b2
y3
b3
y7
b7
y4
b8
y8
b4
y5
b5
y6
b6
b8
y7
b7

Endogenous: PrecursorError(ppm): -6.03 DeNovoScore: 69 MSGFScore: 69 SpecEValue: 9.77E-13 Evalue: 4.04E-10
Synthetic: PrecursorError(ppm): 1.49 DeNovoScore: 86 MSGFScore: 85 SpecEValue: 3.91E-13 Evalue: 1.62E-10

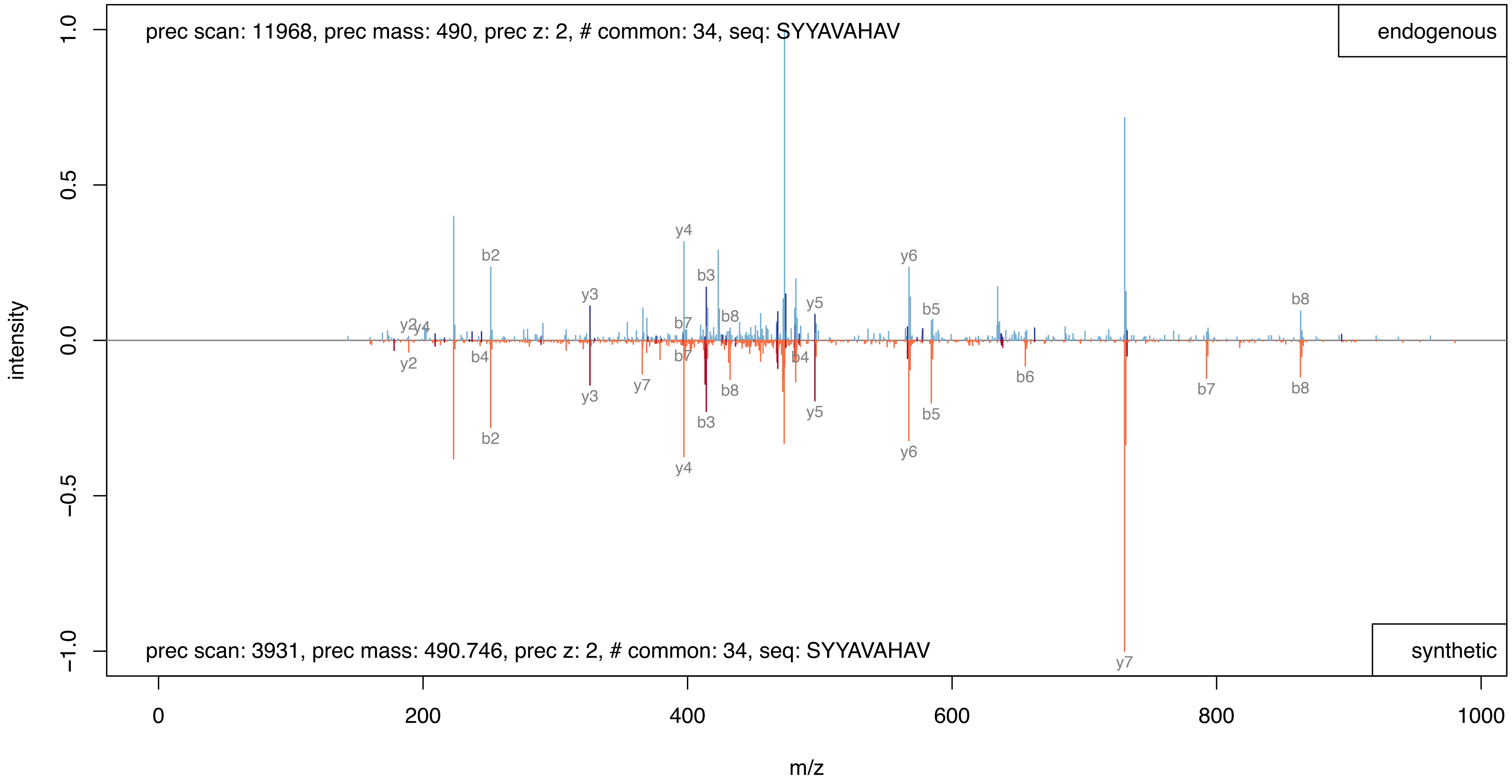

Endogenous: PrecursorError(ppm): 8.95 DeNovoScore: 49 MSGFScore: -27 SpecEValue: 2.36E-03 Evalue: 9.77E-01
Synthetic: PrecursorError(ppm): 0.70 DeNovoScore: 87 MSGFScore: 87 SpecEValue: 3.91E-13 Evalue: 1.62E-10

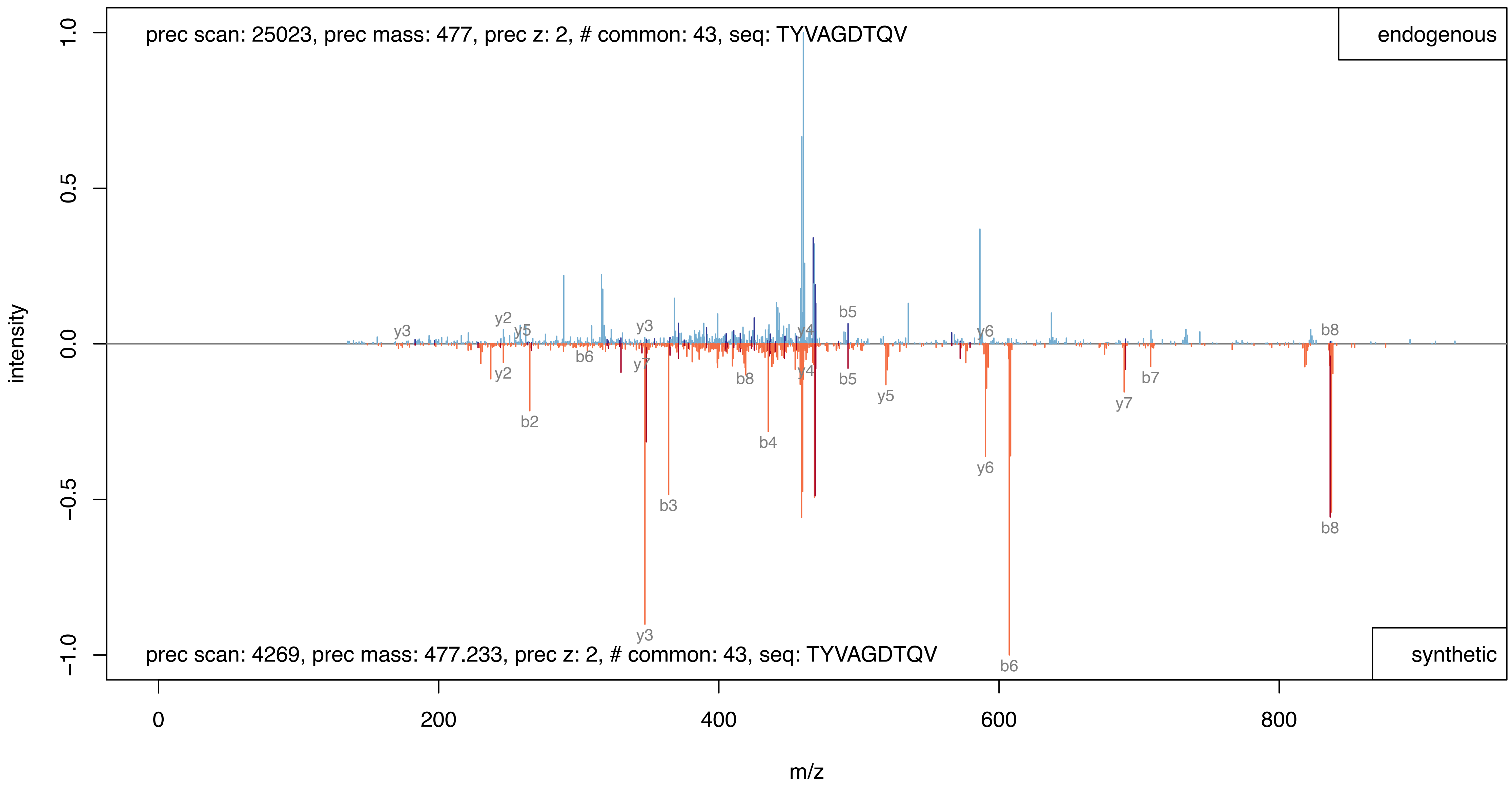

Endogenous: PrecursorError(ppm): -6.47 DeNovoScore: 69 MSGFScore: 42 SpecEValue: 1.27E-09 Evalue: 5.24E-07
Synthetic: PrecursorError(ppm): -0.18 DeNovoScore: 112 MSGFScore: 103 SpecEValue: 1.42E-11 Evalue: 5.88E-09

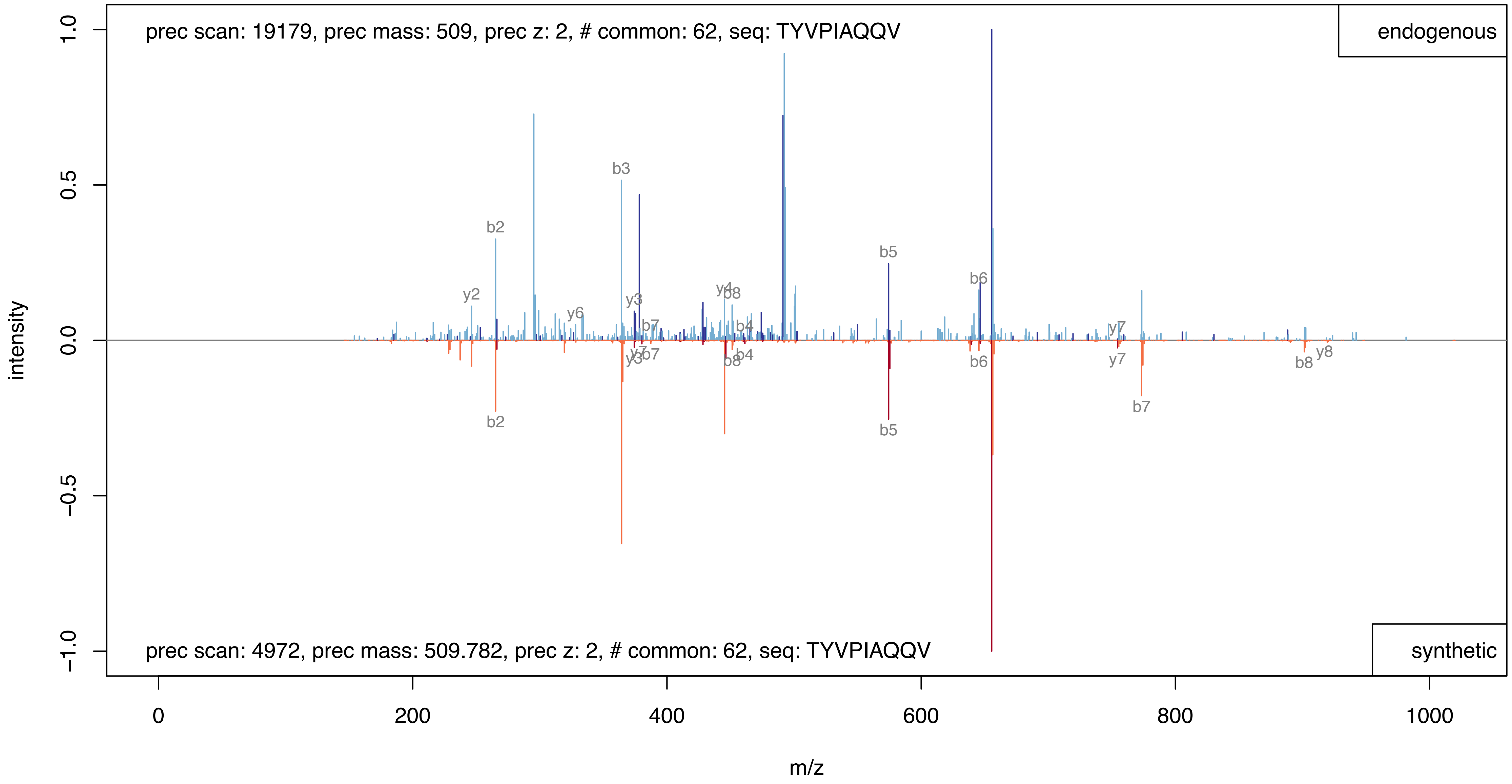

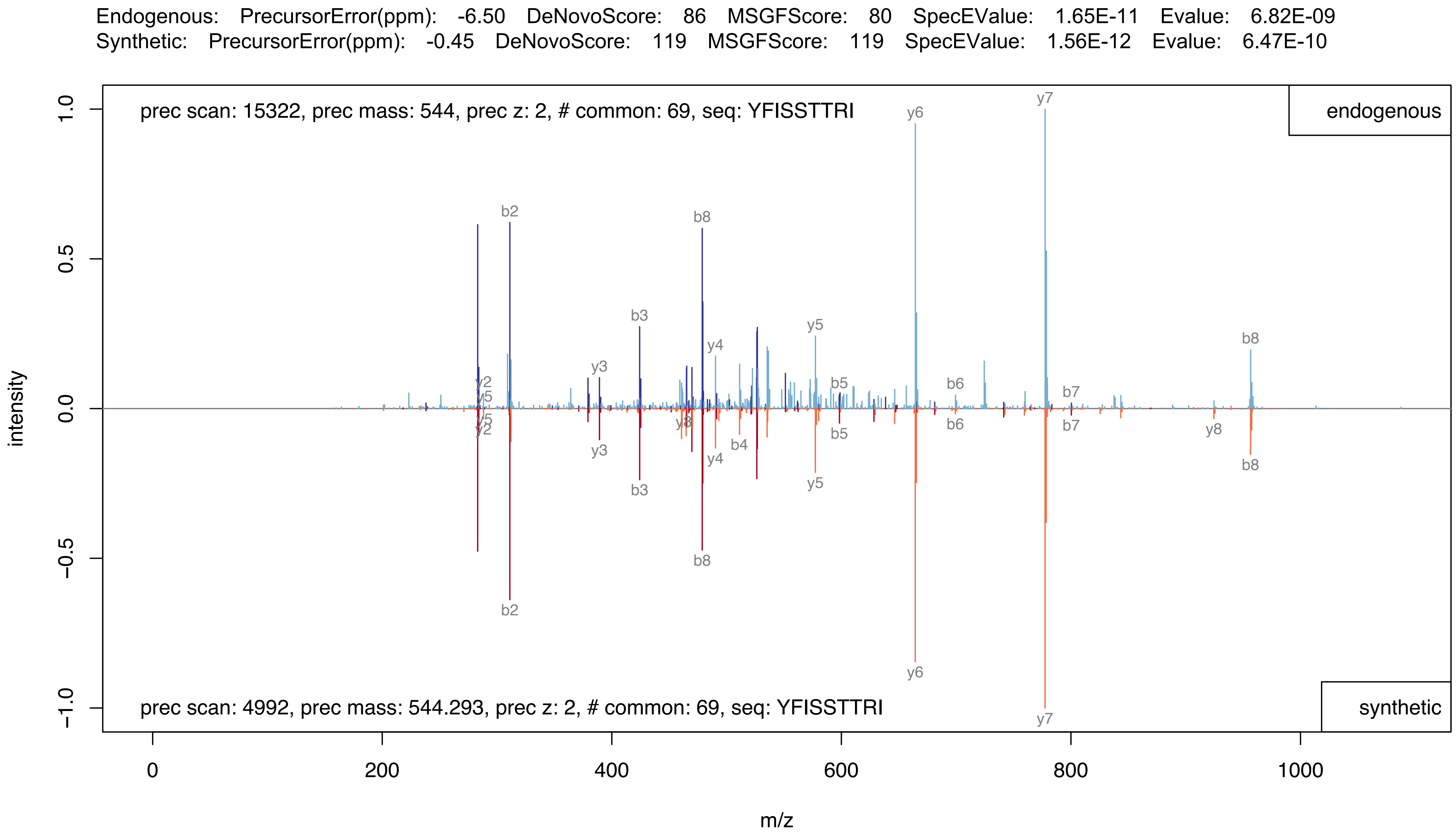
Endogenous: PrecursorError(ppm): -6.50 DeNovoScore: 86 MSGFScore: 80 SpecEValue: 1.65E-11 Evalue: 6.82E-09
Synthetic: PrecursorError(ppm): -0.45 DeNovoScore: 119 MSGFScore: 119 SpecEValue: 1.56E-12 Evalue: 6.47E-10
prec scan: 15322, prec mass: 544, prec z: 2, # common: 69, seq: YFISSTTRI
endogenous
prec scan: 4992, prec mass: 544.293, prec z: 2, # common: 69, seq: YFISSTTRI
synthetic
intensity
m/z
y7
y6
b2
b8
b3
y3
y4
y5
b5
b6
b7
y8
b4
y2
1.0
0.5
0.0
−0.5
−1.0
0
200
400
600
800
1000
